# Transgenic maize phospho*enol*pyruvate carboxylase alters leaf–atmosphere CO_2_ and ^13^CO_2_ exchanges in *Oryza sativa*

**DOI:** 10.1007/s11120-019-00655-4

**Published:** 2019-07-19

**Authors:** Rita Giuliani, Shanta Karki, Sarah Covshoff, Hsiang-Chun Lin, Robert A. Coe, Nuria K. Koteyeva, Marc A. Evans, W. Paul Quick, Susanne von Caemmerer, Robert T. Furbank, Julian M. Hibberd, Gerald E. Edwards, Asaph B. Cousins

**Affiliations:** 1 School of Biological Sciences, Molecular Plant Sciences, Washington State University, Pullman, WA 99164-4236 USA; 2 C4 Rice Center, International Rice Research Institute (IRRI), Los Baños, Philippines; 3 Department of Plant Sciences, University of Cambridge, Cambridge, CB2 3EA UK; 4 Laboratory of Anatomy and Morphology, V.L. Komarov Botanical Institute of the Russian Academy of Sciences, Prof. Popov Street 2, St. Petersburg, Russia 197376; 5 Department of Mathematics and Statistics, Washington State University, Pullman, WA 99164-3113 USA; 6 Department of Animal and Plant Sciences, University of Sheffield, Sheffield, S10 2TN UK; 7 Division of Plant Sciences, Research School of Biology, The Australian National University, Canberra, ACT 0200 Australia

**Keywords:** C_4_ photosynthesis, Leaf ^13^CO_2_ discrimination, Leaf dark respiration, *Oryza sativa*, *PEPC* overexpression, Rice

## Abstract

The engineering process of C_4_ photosynthesis into C_3_ plants requires an increased activity of phospho*enol*pyruvate carboxylase (PEPC) in the cytosol of leaf mesophyll cells. The literature varies on the physiological effect of transgenic maize (*Zea mays*) PEPC (*Zm*PEPC) leaf expression in *Oryza sativa* (rice). Therefore, to address this issue, leaf–atmosphere CO_2_ and ^13^CO_2_ exchanges were measured, both in the light (at atmospheric O_2_ partial pressure of 1.84 kPa and at different CO_2_ levels) and in the dark, in transgenic rice expressing *ZmPEPC* and wild-type (WT) plants. The in vitro PEPC activity was 25 times higher in the *PEPC* overexpressing (*PEPC*-OE) plants (~20% of maize) compared to the negligible activity in WT. In the *PEPC*-OE plants, the estimated fraction of carboxylation by PEPC (*β*) was ~6% and leaf net biochemical discrimination against ^13^CO_2_$\left( {\Delta_{\text{bio}} } \right)$ was ~ 2‰ lower than in WT. However, there were no differences in leaf net CO_2_ assimilation rates (*A*) between genotypes, while the leaf dark respiration rates (*R*_d_) over three hours after light–dark transition were enhanced (~ 30%) and with a higher ^13^C composition $\left( {\delta^{ 1 3} {\text{C}}_{\text{Rd}} } \right)$ in the *PEPC*-OE plants compared to WT. These data indicate that *Zm*PEPC in the *PEPC*-OE rice plants contributes to leaf carbon metabolism in both the light and in the dark. However, there are some factors, potentially posttranslational regulation and PEP availability, which reduce *Zm*PEPC activity in vivo.

**Electronic supplementary material:**

The online version of this article (10.1007/s11120-019-00655-4) contains supplementary material, which is available to authorized users.

## Introduction

Genetic engineering of C_3_ plants to perform C_4_ photosynthesis will require overexpression of phospho*enol*pyruvate carboxylase (PEPC) in the leaf mesophyll (M) cells to catalyze the initial carboxylation reaction (Gehlen et al. 1996; Häusler et al. 2002; Fukayama et al. 2003). To date, the overexpression of *PEPC* (*PEPC*-OE) in M cells of several C_3_ species (e.g., tobacco, potato, wheat, *Arabidopsis thaliana*, and rice) has shown differences in PEPC content, activity, and physiological impacts (Matsuoka et al. 2001; Häusler et al. 2002; Miyao and Fukayama 2003; Zhu et al. 2010; Qi et al. 2017). The presence of transgenic C_4_-type PEPC activity in rice enhanced the stomatal and mesophyll CO_2_ conductances (*g*_sC_ and *g*_m_, respectively) and improved leaf net CO_2_ assimilation rates (Ku et al. 1999, 2000; Lian et al. 2014). Alternatively, other rice *PEPC*-OE studies showed decreases in leaf net CO_2_ assimilation compared to wild-type (WT), particularly under low photorespiratory conditions. This result was suggested to occur because of reduced ribulose-1,5-bisphosphate carboxylase:oxygenase (Rubisco) activity (Fukayama et al. 2003; Taniguchi et al. 2008), and enhanced rates of leaf mitochondrial non-photorespiratory CO_2_ release in the light (*R*_L_) (Miyao 2003). Higher *R*_L_ in the *PEPC*-OE plants is consistent with the anaplerotic function of PEPC (Jeanneau et al. 2002; O’Leary et al. 2011; Abadie and Tcherkez 2018) and a stimulation of the tricarboxylic acid (TCA) cycle with the increased PEPC activity (Agarie et al. 2002; Fukayama et al. 2003; Kandoi et al. 2016). In addition, a decrease in O_2_ sensitivity in PEPC-OE rice plants was reported (Agarie et al. 2002; Fukayama et al. 2003). Collectively, the increase in PEPC activity in C_3_ plants has provided inconsistent effects on leaf photosynthesis, despite indications that the transferred *PEPC* gene can produce a functional enzyme (Fukayama et al. 2003). In addition, subtle changes in leaf CO_2_ assimilation may not be detected with traditional leaf gas exchange measurements.

However, the combined analysis of leaf–atmosphere CO_2_ exchange and discrimination against ^13^CO_2_ can be a useful tool to gain mechanistic insights into changes in leaf carbon metabolism in the light (Farquhar et al. 1989; von Caemmerer 1989; Ghashghaie et al. 2003; Cousins et al. 2007; von Caemmerer et al. 2014). For example, in C_3_ plants (instantaneous) leaf net discrimination against ^13^CO_2_ in the light (∆_o_, ‰) is determined by the discrimination against ^13^CO_2_ during CO_2_ movement from the atmosphere to the chloroplast stroma of M cells, and the ^13^CO_2_ discriminations associated with carboxylation, photorespiration, and mitochondrial non-photorespiratory respiration (von Caemmerer and Evans 1991; Le Roux et al. 2001; Barbour et al. 2010; Bickford et al. 2010; Tazoe et al. 2011; Evans and von Caemmerer 2013). The ^13^CO_2_ fractionation due to the carboxylation reactions (*b*, ‰) includes the contributions of both Rubisco and PEPC (Cernusak et al. 2013; Ubierna and Farquhar 2014), where the ^13^CO_2_ fractionation by Rubisco (*b*_3_) is ~29.0‰ with respect to dissolved CO_2_ (Roeske and O’Leary 1984) and is typically assumed to be fairly constant across species and insensitive to temperature (Ghashghaie et al. 2003; Evans and von Caemmerer 2013). By contrast, the net ^13^CO_2_ fractionation associated with PEPC (*b*_4_) is *circa* − 5.7‰ at 25 °C, which includes the ^13^C fractionations during CO_2_ dissolution in water, catalyzed hydration of CO_2_ to bicarbonate (HCO_3_^−^) and PEP carboxylation (von Caemmerer et al. 2014). In C_3_ plants, *b* for gaseous CO_2_ ranges between 28.2 and 30‰, corresponding to carboxylation by PEPC from 5% of total leaf carbon uptake to zero, respectively (Brugnoli et al. 1988; Gillon and Griffiths 1997; Ghashghaie et al. 2003). When the contribution by PEPC activity to total carbon uptake rate is large, for example, in the C_3_–C_4_ intermediate *Flaveria floridana* where PEPC activity was estimated to be 12 to 20% of the total carboxylation, this can significantly affect ∆_o_ (Alonso-Cantabrana and von Caemmerer 2016). This impact of PEPC activity on ∆_o_ provides a benchmark for evaluating the contribution of C_4_-PEPC (e.g., from *Zea mays*, *Zm*PEPC) activity to carbon uptake in rice.

However, in addition to the carboxylation reactions, photorespiration and mitochondrial non-photorespiratory respiration can also influence ∆_o_. For example, ∆_o_ decreases at a given ratio of intercellular: atmospheric CO_2_ partial pressures (*C*_i_/*C*_a_) with the discrimination against ^13^CO_2_ associated with photorespiration (∆_f_). By contrast, discrimination against ^13^CO_2_ associated with non-photorespiratory mitochondrial respiration (∆_e_) may decrease or increase ∆_o_ depending on the offset in ^13^CO_2_ compositions $\left( {\delta^{ 1 3} {\text{C}},\permille} \right)$ between leaf chamber during measurements and growth chamber (Gillon and Griffiths 1997; Ghashghaie et al. 2003; Gong et al. 2015). The absolute ^13^CO_2_ fractionation due to mitochondrial non-photorespiratory decarboxylating reactions (*e*, ‰; relative to photosynthetic products) is usually considered to be lower than the ^13^CO_2_ fractionation of photorespiration (*f*, ‰; Rooney 1988; Ivlev et al. 1996; Tazoe et al. 2009; Wingate et al. 2007). However, the magnitude of ∆_e_ (‰) depends on *e*, the ^13^CO_2_ signature of the substrates used for respiration (Stutz et al. 2014) and the ratio of *R*_L_ to the carboxylation rate. The leaf net discrimination against ^13^CO_2_ leads to photosynthetic products depleted in the heavier carbon isotope compared to atmospheric CO_2_, and since ∆_o_ and the carbon pool used by leaf respiration change during the day (Tcherkez et al. 2017a), it is difficult to accurately estimate $\delta ^{{13}} {\text{C}}$ of *R*_L_ substrates. In addition, estimates of *R*_L_ are indirect, and it has been shown that mitochondrial respiration activity is partially inhibited in the light (Tcherkez et al. 2005). There are also indications that photorespiration plays a role in regulating the degree of *R*_L_ inhibition (Abadie et al. 2017; Tcherkez et al. 2017a; Gauthier et al. 2018). Alternatively, rates of leaf CO_2_ evolution in the dark (*R*_d_) and $\delta^{ 1 3} {\text{C}}$ of dark-respired CO_2_$\left( {\delta^{ 1 3} {\text{C}}_{\text{Rd}} ,\permille} \right)$ can be directly measured, where $\delta^{ 1 3} {\text{C}}_{\text{Rd}}$ largely depends on the $\delta^{ 1 3} {\text{C}}$ of substrates feeding respiration, particularly the pool of leaf carbon previously produced in the light (Atkin et al. 1998; Barbour et al. 2007; Lehmann et al. 2016a, b; Gessler et al. 2017). Higher *R*_d_ values in PEPC overexpressing plants have been observed on potato (Gehlen et al. 1996; Häusler et al. 1999; Rademacher et al. 2002) and also rice (Agarie et al. 2002). Therefore, combined analysis of the leaf CO_2_ evolution flux in the dark (*R*_d_) and of $\delta^{ 1 3} {\text{C}}_{\text{Rd}}$ may provide useful insights into leaf carbon metabolism in *PEPC*-OE plants versus WT in the light.

The purpose of the current study was to quantify the effects of transgenic expression of *Zm*PEPC on leaf–atmosphere CO_2_ exchange and discrimination against ^13^CO_2_ in *Oryza sativa*, with particular interest on the net biochemical ^13^CO_2_ fractionation due to the carboxylating enzymes (∆_bio_). Measurements were made under low photorespiratory conditions (O_2_ partial pressure of 1.84 kPa) to minimize the contribution of photorespiration and refixation of photorespired CO_2_ to leaf net discrimination against ^13^CO_2_. In addition, three atmospheric CO_2_ partial pressures (*p*CO_2_) were used (18.4, 35.0, and 92.1 Pa) to potentially manipulate the relative contributions of PEPC and Rubisco to carboxylation. Leaf net biochemical discrimination against ^13^CO_2_ (Δ_bio_, ‰), which includes both Rubisco and PEPC fractionations, was used to estimate the in vivo contribution to carboxylation by PEPC (*β*) in the *PEPC*-OE plants. Leaf–atmosphere CO_2_ and ^13^CO_2_ exchanges were also measured in the dark to determine *R*_d_ and $\delta^{ 1 3} {\text{C}}_{\text{Rd}}$ to further explain the effect of *Zm*PEPC to leaf carbon metabolism.

## Materials and methods

### Plant material

#### Generation of ZmPEPC-expressing transgenic rice lines

Generation of transgenic *Oryza sativa* (rice) lines expressing maize (*Zea mays*) *phospho*enol*pyruvate carboxylase* (*ZmPEPC*) was done at the International Rice Research Institute (IRRI; Los Baños, Philippines). *Agrobacterium tumefaciens* (strain LBA4404)-mediated transformation was performed following the method described by Hiei and Komari (2006). A pSC0/*ZmPEPC* vector (see Supplementary material, Fig. S1A) containing a full-length genomic fragment (GenBank Accession no. AF234296.1) was created by subcloning *ZmPEPC* from pIG121Hm/*ZmPEPC* (from Mitsue Miyao, National Institute of Agrobiological Sciences, Tsukuba, Japan; Ku et al. 1999) into pSC0 (GenBank, Accession no. KT365905; Lin et al. 2016) using a SmaI restriction digest. Expression of *ZmPEPC* is driven by its native promoter and terminator. A co-transformation of pSC0/*ZmPEPC* with pCAMBIA1300, a binary vector with the hygromycin B resistance gene, was performed for selection. Freshly harvested immature embryos of rice (*Oryza sativa* spp. *indica* cv. IR64) 8–12 days after anthesis were used as explants. After one week of co-cultivation in Murashige and Skoog medium and resting for 5 days, emerging resistant calli were selected with 50 mg L^−1^ of hygromycin B. A total of 83 transgenic rice plantlets regenerated from hygromycin-resistant calli were kept in hydroponics (Yoshida culture solution; Yoshida et al. 1972) for 2 weeks to acclimate. A total of 59 *ZmPEPC* PCR positive plants from these lines were grown in soil. Plants with a single copy of the transgene and more than 50% *Zm*PEPC protein accumulation relative to the maize control were advanced to further generations to obtain homozygous lines. At T_3_ generation, homozygosity was confirmed on four plants (events) by DNA blot analysis for T-DNA integration (see Fig. S1B for PEPC-28 event). All four events had abundant *Zm*PEPC accumulation relative to maize as detected by immunoblot (see Fig. S2A). Progeny of the PEPC**-**28 event showed that detectable *Zm*PEPC localizes to rice M cells (Fig. S2B), and the subsequent T_4_ generation (line PEPC-28) was chosen for analysis in the present study. *Oryza sativa* cv. IR64 line A009 was used as a negative control for transgene expression, and *Zea mays* cv. B73 was used as a positive control for protein accumulation throughout.

#### Plant growth conditions

*PEPC*-OE (line PEPC-28) and WT plants (cv. IR64, line A009) were grown, together with *Zea mays* cv. B73 plants, in a controlled environment growth chamber (G_ch_; Bigfoot series, BioChambers Inc., Winnipeg, MB, Canada) at the School of Biological Sciences at Washington State University, Pullman, WA (USA). Plants were individually grown in 4-L free drainage pots as described in Giuliani et al. (2013). The photoperiod was 14 h, from 8:00 to 22:00 h standard time. Light was provided by F54T5/841HO Fluorescent 4100 K and 40 W Halogen incandescent bulbs (Philips) and was supplied in a bell**-**shaped pattern during the photoperiod with a maximum photosynthetic photon flux density (PPFD) of 600 mol photons m^−2^ s^−1^ incident on the plant canopy for 10 h. Air temperature was 22 °C in the dark, and after switching on the light, it tracked the PPFD pattern with a maximum of 26 °C for 10 h. Air relative humidity was maintained at ~70% so that the maximum air Vapor Pressure Deficit (VPD) was ~1.6 kPa. During the photoperiod, the atmospheric *p*CO_2_ in the G_ch_ was enriched with CO_2_ supplied by a pressurized tank and maintained at 184.2 Pa (2000 *μ*mol CO_2_ mol^−1^ air); the ^13^CO_2_ composition $\left( {\delta^{ 1 3} {\text{C}}_{\text{Gch}} } \right)$ was − 41.6‰ (± 0.1 SE; *n *= 8) determined as in Giuliani et al. (2019).

### Leaf biochemical analysis

The percentage of PEPC in *PEPC*-OE and WT rice compared to maize was determined by protein immunoblot technique according to Koteyeva et al. (2015). In the two rice plant types and in maize, the in vitro activities of PEPC and Rubisco per unit leaf surface area (μmol m^−2^ s^−1^) were determined spectrophotometrically as described by Cousins et al. (2007) and Walker et al. (2013), respectively. Leaf malate content (mmol malate m^−2^) was determined spectrophotometrically based on the method of Hatch (1979) and Edwards et al. (1982). These methods for leaf biochemical analysis are reported in Method S1.

### Leaf physiological analysis

#### System set-up for coupled measurements of leaf–atmosphere CO_2_, H_2_O, and ^13^CO_2_ exchanges

Measurements were performed in Pullman, WA (mean atmospheric pressure of 92.1 kPa). Two LI**-**6400XT portable gas analyzers (LI**-**COR Biosciences, NE, USA; detecting ^12^CO_2_) operating as an open system were coupled to a tunable**-**diode laser absorption spectroscope, which detects ^12^CO_2_ and ^13^CO_2_ isotopologs (TDLAS model TGA200A, Campbell Scientific, Inc., Logan, UT, USA). The system set-up was as described in Giuliani et al. (2019), based on Ubierna et al. (2013), Stutz et al. (2014), and Sun et al. (2014).

Leaf photosynthesis was determined with a LI**-**COR equipped with a 2 × 3 cm^2^ leaf chamber (*L*_ch_) and a 6400**-**02B LED light source (LI**-**COR Biosciences). Alternatively, leaf dark respiration was determined with an 8 × 10 cm^2^ custom**-**built L_ch_ having an adaxial glass window, and with a volume of ~ 100 cm^3^ (Barbour et al. 2007, based on Sharkey et al. 1985). The chamber had a hollowed stainless-steel frame sealed with a closed**-**cell foam gasket and was connected to a circulating water bath for temperature control in the lumen. Before the dark respiration measurements, the leaf portion in the L_ch_ was illuminated with a 6400**-**18 RGB light source (LI**-**COR Biosciences).

#### Protocol for coupled measurements of leaf–atmosphere CO_2_, H_2_O, and ^13^CO_2_ exchanges

Leaf photosynthetic measurements (*n *= 4 in *PEPC*-OE and *n* = 5 in WT) were taken between 9:00 and 16:00 h standard time; on each plant, the mid-to-distal portions of two fully expanded leaves from the same stem were positioned to completely cover the *L*_ch_ section. Measurements were taken at atmospheric CO_2_ partial pressures in the *L*_ch_ (*C*_a_) of 18.4, 35.0, and 92.1 Pa (i.e., 200, 380, and 1000 µmol CO_2_ mol^−1^ air, respectively) and with ^13^CO_2_ composition entering the *L*_ch_ (*δ*_in_, corresponding to the ^13^C composition of the CO_2_ source from a pressurized tank) of − 48.0‰. The O_2_ partial pressure (*p*O_2_) was set at 1.84 kPa (i.e., 20 mmol O_2_ mol^−1^ air), PPFD was 1500 µmol photons m^−2^ s^−1^, *t*_leaf_ was 25 °C, and leaf**-**to**-**air VPD was kept in the range of 1.0**–**1.5 kPa. The airflow rate through the LI-COR system was 300 µmol s^−1^ (~ 0.48 L min^−1^), and gas analyzers were matched after each change in *C*_a_ when the TDLAS was not measuring the air leaving the *L*_ch_. Leaves were acclimated for about 30 min, and the data were recorded for an additional 30–40 min under each measurement condition. The rate of net CO_2_ assimilation per leaf surface area (*A*, µmol CO_2_ m^−2^ s^−1^), stomatal conductance to CO_2_ diffusion (*g*_sC_, µmol CO_2_ m^−2^ s^−1^ Pa^−1^), intercellular *p*CO_2_ (*C*_i_, Pa), and the ratio *C*_i:_*C*_a_ (*C*_i_/*C*_a_) were determined. The ^13^C signature of leaf dry matter (*δ*^13^C_dm_, ‰) and total N content as fraction (%) of leaf dry matter (*n* = 4 for *PEPC*-OE; *n* = 5 for WT) were determined by isotope ratio mass spectrometry (IRMS) as described in Giuliani et al. (2019). Total N content per unit leaf surface area (g m^−2^) was then calculated based on leaf dry matter per area.

Leaf dark respiration measurements were performed on two plants per day (one *PEPC*-OE and one WT). Each plant was taken out of the G_ch_ at 9:30 h standard time, and the mid-to-distal portions of 8–9 fully expanded leaves, similar to those used for the photosynthetic analysis, were enclosed in the custom**-**built *L*_ch_ to completely cover the section area of ~ 76 cm^2^. Under *p*O_2_ of 1.84 kPa, the leaves were first exposed to 750 µmol photons m^−2^ s^−1^ of PPFD for 20 min, 500 for 15 min (at *t*_leaf_ of 25 °C), and 100 µmol photons m^−2^ s^−1^ for 5 min (at *t*_leaf_ of 30 °C). The airflow rate through the LI-COR system was changed from 700, to 500, and to 350 µmol s^−1^ tracking the decreasing PPFD. The measurement CO_2_ (supplied by a new cartridge every day) had $$\delta^{ 1 3} {\text{C}}$$ from − 6.2 to − 4.8‰ to generate a large $$\delta^{ 1 3} {\text{C}}$$ difference between the *L*_ch_ and the *G*_ch_ atmosphere (− 41.6‰). The *C*_a_ in the *L*_ch_ was maintained at 35.0 Pa. The stepwise decrease in PPFD and airflow rate minimized the perturbation to the gas exchange measurements when transitioning the leaf from the light to dark. After 40 min of leaf photosynthesis, dark was imposed in the L_ch_, and leaf CO_2_ evolution was measured at *p*O_2_ of 18.4 kPa and *t*_leaf_ of 30 °C for 195 min to determine the dynamics of the dark respiration rate per unit (one side) leaf surface area (*R*_d_, µmol CO_2_ m^−2^ s^−1^) and corresponding $$\delta^{ 1 3} {\text{C}}$$$$\left( {\delta^{ 1 3} {\text{C}}_{\text{Rd}} ,\permille} \right)$$ (*n *= 4). The *t*_leaf_ was set at 30 °C to enhance the precision of the dark measurements, and the gas analyzers were matched at the beginning of the dark period and every 15 min thereafter, when the TDLAS was not measuring the leaf chamber air. In addition, three plants (*n *= 3) of the *PEPC*-OE line and of WT were taken out of the G_ch_ at 12:00 h 3 days after their use for photosynthesis measurements and darkened at 25 °C for 24 h. Subsequently, leaf dark CO_2_ evolution was measured at *t*_leaf_ of 30 °C and at *p*O_2_ of 18.4 Pa to determine *R*_d(24h)_ (µmol CO_2_ m^−2^ s^−1^) and $$\delta^{ 1 3} {\text{C}}_{{{\text{Rd}}( 2 4 {\text{h}})}} \left( \permille \right)$$. The description of the abbreviations, and symbol and unit of the environmental parameters and leaf variables are listed in Table S1.

#### The net discrimination against ^13^CO_2_ in the light, mesophyll CO_2_ conductance, and ^13^C composition of dark-evolved CO_2_

Instantaneous leaf net discrimination against ^13^CO_2_ in the light (∆_o_, ‰) was calculated by mass balance according to Evans et al. (1986). The leaf net biochemical discrimination against ^13^CO_2_ (∆_bio_, ‰), which depends on the biochemistry of net CO_2_ uptake, was determined for the *PEPC*-OE plants at the different *C*_a_ (using Eq. S1 in Method S2), where the mesophyll conductance to CO_2_ diffusion (*g*_m_, μmol CO_2_ m^−2^ s^−1^ Pa^−1^) is a required input (Alonso-Cantabrana and von Caemmerer 2016). In the applied procedure, ∆_bio_ and *g*_m_ are not independent variables), and ∆_bio_ is a proxy of *b* (the in vivo ^13^CO_2_ carboxylation fractionation; see Eq. 1 below) that is necessary to determine *g*_m_. The ∆_bio_ in *PEPC*-OE plants were therefore calculated assuming WT *g*_m_ values, which were estimated according to Evans and von Caemmerer (2013) as described in Method S3. Specifically, the mean *g*_m_ values determined on the WT plants at *C*_a_ of 18.4, 35.0, and 92.1 Pa were used to calculate ∆_bio_ (*n *= 4) in *PEPC*-OE plants at the three *C*_a_ values. The assumption of equal *g*_m_ between *PEPC*-OE and WT plants was supported by the sensitivity analysis of ∆_bio_ on *g*_m_ (see Fig. S3) compared with the ∆_bio_ analysis in Alonso-Cantabrana and von Caemmerer (2016) for a C_3_–C_4_ intermediate species. In addition, comparable *g*_m_ values were determined on the *PEPC*-OE and WT plants from measurements of leaf–atmosphere oxygen (in alternative to carbon) isotope exchange (see Table S2; based on Ubierna et al. 2017; Sonawane and Cousins 2018). However, leaf ^18^O based *g*_m_ is not strictly associated with the biochemistry of photosynthesis as is the ^13^C based *g*_m_, and therefore, it could not be used in the present analysis to determine ∆_bio_ in *PEPC*-OE plants.

In *PEPC*-OE plants, the fraction of carboxylation by PEPC (*β*, mol C_(by_PEPC)_ mol^−1^ C_(by_Rubisco+PEPC)_; *n *= 4) was determined by solving for the *β* value that minimized the difference between the ∆_bio_ determined by eq. S1 and ∆_bio_ modeled (∆_bio_mod_, ‰) based on Griffiths et al. (2007) as

1 $$\Delta _{{{\text{bio}}\_{\text{m}}od}} = \left[ {b_{3} - \beta \left( {b_{3} - b_{4} } \right)} \right] - {\text{ }}\frac{{R_{{\text{L}}} e^{*} }}{{\left( {A + R_{{\text{L}}} } \right)}}$$

assuming the ^13^CO_2_ fractionation of Rubisco (*b*_3_) and PEPC (*b*_4_) were 29.0 and − 5.7‰, respectively. The *R*_L_ (µmol CO_2_ m^−2^ s^−1^) is mitochondrial non-photorespiratoy respiration rate in the light; e* (‰) is the experimental ^13^CO_2_ fractionation associated with *R*_L_, and the term (*A *+ *R*_L_) corresponds to the carboxylation rate by Rubisco plus PEPC. Under the assumption that at the same *t*_leaf_, there was no difference between leaf mitochondrial respiration rate in the light (*R*_L_) and three hours after light–dark transition (*R*_d(3h)_, µmol CO_2_ m^−2^ s^−1^; Table 3), *R*_L_ at *t*_leaf_ of 25 °C was predicted from *R*_d(3h)_ at 30 °C using the temperature response function in Bernacchi et al. (2001). The e* (− 6.4‰) was determined as the difference between $\delta^{ 1 3} {\text{C}}$ of the CO_2_ entering the leaf chamber during photosynthetic measurements (− 48‰) and in the growth chamber (− 41.6‰).

The term $\left[ {b_{3} - \beta \left( {b_{3} - b_{4} } \right)} \right]\varvec{ }$ in Eq. 1 is equivalent to the in vivo ^13^CO_2_ carboxylation fractionation (*b*, ‰), as reported in Farquhar and Richards (1984). Therefore, *b* can be calculated for the *PEPC*-OE plants using *β* estimated as described above. Alternatively, ∆_bio_mod_ can be predicted in WT assuming *b* is equal to *b*_3_ (29.0‰), i.e., no *b*_4_ and *β* values were applied given a negligible in vitro PEPC activity in WT plants (see “Results”).

Leaf net discrimination against ^13^CO_2_ in the light for *PEPC*-OE and WT plants was predicted based on Ubierna and Farquhar (2014) with a simplified equation as

2 $$\Delta ^{{13}} {\text{C}}_{{\bmod }} = a + {\mkern 1mu} \left( {b - a} \right) \times C_{{\text{c}}} /C_{{\text{a}}},$$

where *a *= 4.4‰ is the ^13^CO_2_ fractionation during CO_2_ diffusion through stomata; *b* = [*b*_3_ − *β*(*b*_3_–*b*_4_)] for *PEPC*-OE (Eq. 1; see Results) and *b *= 29‰ for WT; *C*_c_ is the *p*CO_2_ in the chloroplast (Pa), as calculated by Fick’s first law as *C*_c_ = *C*_i_ − *A*/*g*_m_.

The ^13^C composition in leaf respired CO_2_ in the dark $(\delta^{ 1 3} {\text{C}}_{\text{Rd}} ,\permille)$ was calculated according to Barbour et al. (2007), based on Evans et al. (1986).

Given $$\delta^{ 1 3} {\text{C}}_{{{\text{Rd}}(i)}}$$ as the value of $$\delta^{ 1 3} {\text{C}}$$ for dark-evolved CO_2_ at time *i* (by 3 min over 195 min from light–dark transition), the fractional contribution of *L*_ch_ carbon assimilates to $$\delta^{ 1 3} {\text{C}}_{{{\text{Rd}}(i)}}$$$$\left( {^{{\delta {\text{Rd}}}} L_{{{\text{ch}}\_{\text{substr}}(i)}} ,{\permille \mathord{\left/ {\vphantom {\permille \permille}} \right. \kern-0pt} \permille}} \right)$$ was calculated for *PEPC*-OE and WT plants (*n* = 4) according to Giuliani et al. (2019) as

3 $${}^{{^{{\delta {\text{Rd}}}} }}{\text{L}}_{{{\text{ch}}\_{\text{substr}}\left( i \right)}} = \frac{{\left( {\delta^{13} C_{{{\text{Rd}}\left( i \right)}} - \delta^{13} C_{{{\text{Rd}}\left( {24{\text{h}}} \right)}} } \right)}}{{\left( {\delta^{13} C_{{{\text{Lch}}\_{\text{Ph}}}} - \delta^{13} C_{{{\text{Rd}}\left( {24{\text{h}}} \right)}} } \right)}},$$

where $\delta^{13} C_{{{\text{Rd}}\left( {24{\text{h}}} \right)}}$ are as given in Table 3, and $\delta^{13} C_{{{\text{Lch}}\_{\text{Ph}}}} \left( \permille \right)$ are the representative $\delta^{ 1 3} {\text{C}}$ of *PEPC*-OE or WT carbon assimilates produced in the L_ch_ before light–dark transition. The assumptions underlying Eq. 3 are described in Giuliani et al. (2019), and the values of the variables used for calculations are presented in Table S3. Based on the total fractional contributions of *L*_ch_ and *G*_ch_ assimilates to $\delta^{ 1 3} {\text{C}}_{\text{Rd}}$ equal 1, the complementing fractional contribution of G_ch_ assimilates to $\delta^{ 1 3} {\text{C}}_{{{\text{Rd}}(i)}} \left( {^{{\delta {\text{Rd}}}} {\text{G}}_{{{\text{ch}}\_{\text{substr}}(i)}} , \, {\permille \mathord{\left/ {\vphantom {\permille \permille}} \right. \kern-0pt} \permille}} \right)$ was determined for both plant types as ${}^{{^{{\delta {\text{Rd}}}} }}{\text{G}}_{{{\text{ch}}\_{\text{substr}}\left( i \right)}} = 1 - {}^{{^{{\delta {\text{Rd}}}} }}{\text{L}}_{{{\text{ch}}\_{\text{substr}}\left( i \right)}}$.

#### Leaf A–C_i_ response curves

For *PEPC*-OE and WT plants, *A*-*C*_i_ response curves (*n* = 4) were determined with the LI**-**6400XT through stepwise decreases in *C*_a_ from 35.0 to 3.7 Pa, at 1.84 kPa *p*O_2_, PPFD of 1500 µmol photons m^−2^ s^−1^, *t*_leaf_ at 25 °C, and VPD between 1.0 and 1.5 kPa. For each response curve, a least square regression analysis was applied to the initial slope (for *C*_i_ ≤ 9.2 Pa) to calculate the CO_2_ compensation point (*Γ*, Pa).

### Statistical analysis

Statistical analyses for the effects of plant-type (*PEPC*-OE and WT) and/or *C*_a_ level on the leaf photosynthetic and dark respiration variables are described in Method S4. In addition, a nonlinear model with three parameters was used to fit the *R*_d_ and $\delta^{ 1 3} {\text{C}}_{\text{Rd}}$ responses for the two plant types over a 3-hour interval. The significance between the two plant types for the *R*_d_ or $\delta^{ 1 3} {\text{C}}_{\text{Rd}}$ responses was inferred from the analysis of the model parameters, i.e., range (difference between the initial value and the lower asymptote), exponential rate of change, and lower asymptote (floor of the response) (see Methods S4 for the description of the procedure).

## Results

### Leaf biochemical analysis

#### PEPC content and activity

**Fig. 1:**
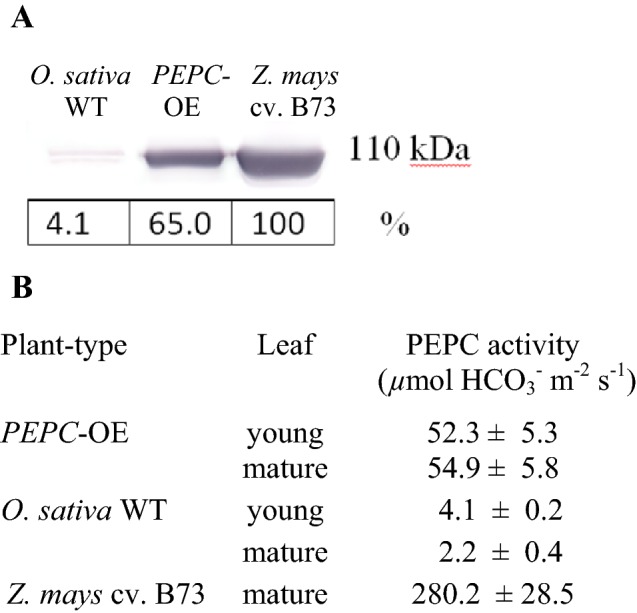
**a** Immunoblot analysis for PEPC from soluble proteins extracted from mature rice leaves, showing protein molecular weight (kDa) and band intensity quantitation. The levels of PEPC for both *PEPC*-OE and WT are mean percentage values of *Z. mays* (*n *=2). **b** In vitro PEPC activity determined in both young and mature leaves of *PEPC*-OE and WT, and mature leaves of *Z. mays* plants. Values are mean ± SE (*n *= 3)

Mean PEPC content in *PEPC*-OE and WT plants compared to *Z. mays* were 65% ± 2.2 SE and 4.1% ± 0.4 SE, respectively (Fig. 1a; *n *= 2). The in vitro mean activities of PEPC in young (expanding) leaves of *PEPC*-OE and WT plants were 52.3 and 4.1 µmol HCO_3_^−^ m^−2^ s^−1^, respectively, and 54.9 and 2.2 µmol HCO_3_^−^ m^−2^ s^−1^ in mature leaves, respectively. The in vitro mean activity of PEPC in mature *Z. mays* leaves was 280 µmol HCO_3_^−^ m^−2^ s^−1^, and thus the PEPC activity in *O. sativa PEPC*-OE mature leaves was approximately 25 times greater than WT but ~ 5 times lower than that in *Z. mays* (Fig. 1b; *n *= 3). The mean ratios of in vitro PEPC: Rubisco activity in mature leaves were 0.79 ± 0.07 SE and 0.04 ± 0.01 SE for *PEPC*-OE and WT plants, respectively (*n *= 3).

#### Leaf malate content

The mean malate contents per unit leaf surface area (mmol malate m^−2^) determined on leaf samples taken immediately after leaf photosynthetic measurements were 0.60 ± 0.13 SE in *PEPC*-OE and 0.55 ± 0.06 SE in WT plants, but not statistically different between plant types (*n *= 5; *P* > 0.05).

### Leaf physiological analysis

#### Leaf photosynthetic responses

There was a significant *p*CO_2_ effect on *A*, *g*_sC_, *C*_i_, *C*_i_/*C*_a_, *C*_c_, *C*_c_/*C*_a_, and ∆_o_, but these parameters did not differ between *PEPC*-OE and WT plants (Table 1). In WT plants, *g*_m_ significantly decreased with *p*CO_2_ (Table 2).

**Table 1:** Leaf photosynthetic traits of *PEPC*-OE (*n *= 4) and WT (*n *= 5) plants at different atmospheric *C*_a_ and *p*O_2_ of 1.84 kPa

Plant-type	*C*_a_ (Pa)	*A* (µmol CO_2_ m^−2^ s^−1^)	*g*_sC_ (µmol CO_2_ m^−2^ s^−1^ Pa^−1^)	*C*_i_ (Pa)	*C*_i_*/C*_a_	*C*_c_ (Pa)	*C*_c_*/C*_a_	∆_o_ (‰)
*PEPC*-OE	18.4	14.4 ± 0.5	2.05 ± 0.17	10.3 ± 0.3	0.56 ± 0.02	7.0 ± 0.2	0.38 ± 0.01	12.8 ± 0.4
	35.0	27.0 ± 0.6	2.42 ± 0.14	21.9 ± 0.5	0.62 ± 0.01	15.4 ± 0.5	0.44 ± 0.01	14.7 ± 0.9
	92.1	35.8 ± 1.2	1.89 ± 0.32	68.2 ± 3.0	0.74 ± 0.03	54.1 ± 2.6	0.59 ± 0.03	17.8 ± 1.1
WT	18.4	15.7 ± 0.9	2.32 ± 0.17	10.6 ± 0.3	0.57 ± 0.01	6.9 ± 0.2	0.38 ± 0.01	13.3 ± 0.3
	35.0	26.5 ± 1.9	2.41 ± 0.33	21.6 ± 0.8	0.62 ± 0.02	15.0 ± 1.0	0.43 ± 0.03	14.8 ± 0.7
	92.1	35.2 ± 1.3	1.40 ± 0.09	63.3 ± 1.0	0.69 ± 0.01	49.2 ± 0.6	0.53 ± 0.01	17.4 ± 0.2
Significance	Plant-type	*P* = 0.953	*P* = 0.697	*P* = 0.176	*P* = 0.460	*P* = 0.277	*P* = 0.201	*P* = 0.914
	CO_2_ level	*P* < 0.001	*P* = 0.012	*P* < 0.001	*P* < 0.001	*P* < 0.0001	*P* < 0.0001	*P* < 0.001
	POC Linear	ns	*P* = 0.009	*P* < 0.001	*P* < 0.001	*P* < 0.0001	*P* < 0.0001	*P* < 0.001
	POC Quadratic	*P* < 0.001	ns	ns	ns	ns	ns	ns
	Plant-type ×CO_2_ level	*P* = 0. 708	*P* = 0.262	*P* = 0.091	*P* = 0.149	*P* = 0.178	*P* = 0.417	*P* = 0.788

*A* is leaf net CO_2_ assimilation rate, *g*_sC_ is stomatal conductance to CO_2_ diffusion, *C*_i_ is *p*CO_2_ in the intercellular air space, *C*_c_ is chloroplast *p*CO_2_, ∆_o_ is leaf net discrimination against ^13^CO_2_ in the light. In *PEPC*-OE plants, *C*_c_ was calculated based on the mesophyll conductance to CO_2_ diffusion (*g*_m_) values used for ∆_bio_ analysis, that is, the *g*_m_ values determined on WT. Values are mean ± SE. Significance (*P* < 0.05) of the effects of plant-type, CO_2_ level, and plant-type × CO_2_ level interaction were evaluated by SAS PROC MIXED. The significance of CO_2_ levels was evaluated in terms of linear and quadratic polynomial orthogonal contrasts (POC)

**Table 2:** Leaf mesophyll CO_2_ conductance (*g*_m_) values of WT plants (*n *= 5) at different atmospheric *C*_a_, which were applied also to *PEPC*-OE plants for ∆_bio_ analysis

	WT			Significance
*C*_a_(Pa)	18.4	35.0	92.1	
*g*_m_ (µmol CO_2_ m^−2^ s^−1^ Pa^−1^)	4.4 ± 0.4	4.2 ± 0.6	2.6 ± 0.2	*P* = 0.016

Values are mean ± SE. Significance (*P* < 0.05) of the effect of CO_2_ level was evaluated by SAS PROC MIXED

**Fig. 2:**
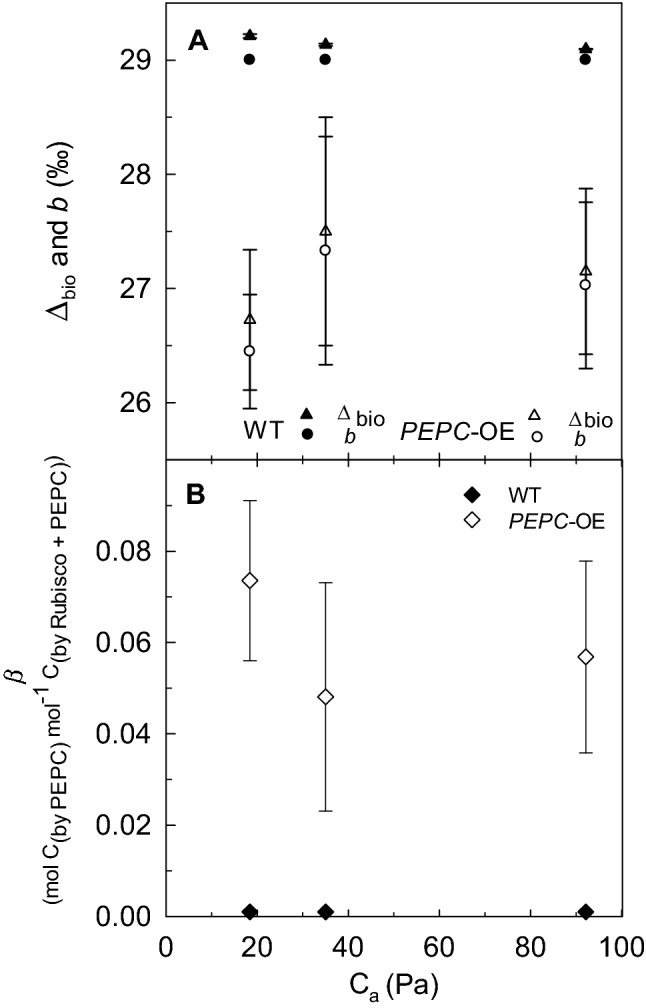
**a** The net ^13^C biochemical discrimination (∆_bio_; triangles) and the in vivo ^13^CO_2_ carboxylation fractionation (*b*; circles) in the *PEPC*-OE (empty symbols) and WT (full symbols) plants at *C*_a_ of 18.4, 35.0, and 92.1 Pa. For WT plants ∆_bio_ = ∆_bio_mod_ and *b* equal to 29.0‰. **b** The carboxylation by PEPC (*β*) in *PEPC*-OE plants (empty symbol) compared to *β* equal to zero in WT (full symbol) at *C*_a_ of 18.4, 35.0, and 92.1 Pa. In both plots, symbols correspond to mean value ± SE (for *PEPC*-OE: *n *= 3 at *C*_a_ of 35.0 Pa, *n *= 4 at *C*_a_ of 18.4 and 92.1 Pa; *n *= 4 for WT)

A significant plant-type effect was determined on ∆_bio_ (‰), with lower values in *PEPC*-OE plants with respect to WT (Fig. 2a; *P* = 0.006), and on *b* (‰), with lower values in the *PEPC*-OE plants compared to a *b* of 29.0 in WT plants (Fig. 2a; *P* = 0.003). In addition, the fraction of carboxylation by PEPC (*β*) in the *PEPC*-OE plants was significantly different from *β* equal to 0 in WT (Fig. 2b; *P* < 0.001). The values of ∆_bio_ and *b* tended to be lower, and *β* values greater, at *C*_a_ of 18.4 Pa compared to the higher *p*CO_2_, but there was not a significant effect of *p*CO_2_ or a plant-type × *p*CO_2_ effect on these parameters. For *PEPC*-OE and WT plants (*n* = 4), the overall means of ∆_bio_ across the three experimental *p*CO_2_ range were 27.1 ± 0.5 SE and 29.2 ± 0.5 SE, respectively. In addition, *b* overall mean of 26.9 ± 0.5 SE and *β* overall mean of ~ 0.06 mol C_(by_PEPC)_ mol^−1^ C_(by_Rubisco+PEPC)_ ± 0.01 SE were calculated for the *PEPC*-OE plants.

**Fig. 3:**
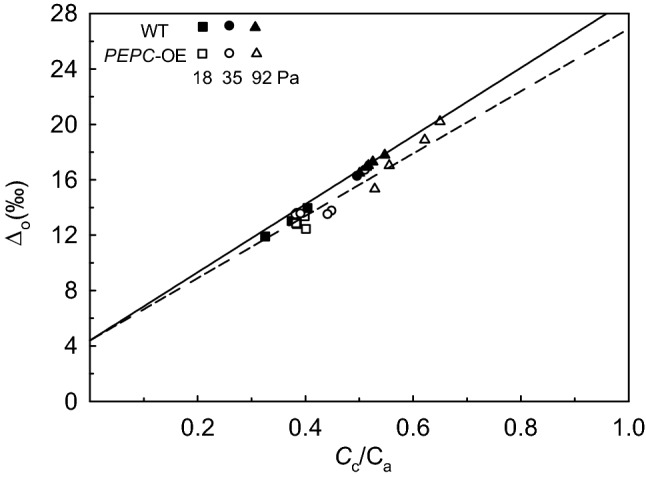
Leaf net discrimination against ^13^CO_2_ in the light (∆_o_) versus ratio of chloroplast to atmosphere CO_2_ partial pressures (*C*_c_/*C*_a_) determined on individual *PEPC*-OE and WT plants (*n *= 4) at *C*_a_ of 18.4, 35.0, and 92.1 Pa under *p*O_2_ of 1.84 kPa. Closed symbols are for WT, and open symbols are for *PEPC*-OE plants; squares for *C*_a_ = 18.4, circles for *C*_a_ = 35.0 and triangles for *C*_a_ = 92.1 Pa. Lines represent the leaf net discrimination against ^13^CO_2_ predicted as ∆^13^C_mod_ = *a *+ (*b*-*a*) × *C*_c_/*C*_a_ (based on Ubierna and Farquhar 2014) where *b *= 29.0‰ for WT (solid line) and *b *= 26.9‰ for *PEPC*-OE (dashed line); *a* equal to 4.4‰

The ∆^13^C_mod_ values for WT and *PEPC*-OE plants significantly fit the corresponding ∆_o_ values plotted versus *C*_c_/*C*_a_, with slopes of 24.6‰ in WT (*R*^2^ = 0.93; *P* < 0.001) and 22.5‰ (*R*^2^ = 0.87; *P* < 0.001) in *PEPC*-OE plants (Fig. 3). The ∆_o_ plotted *versus C*_c_/*C*_a_ was generally higher for the WT compared to *PEPC*-OE plants (Fig. 3), and the regression line fitting ∆_o_ in the two plant types showed a significantly higher slope in WT (24.8‰) compared to *PEPC*-OE (23.1‰) plants (*P* < 0.001; See Method S4 for statistical analysis).

There was not a significant plant-type effect on $\delta^{ 1 3} {\text{C}}$ of leaf biomass ($\delta^{ 1 3} {\text{C}}_{\text{dm}}$; Table 3); in addition, leaf nitrogen content (g m^−2^) was not statistically different between *PEPC*-OE and WT plants: 2.0 ± 0.2 SE and 2.5 ± 0.2 SE (*n *= 4), respectively. Furthermore, the CO_2_ compensation point ($\varGamma$, Pa) was not significantly different between transgenic and WT plants, with mean values of 0.70 ± 0.09 SE and 0.61 ± 0.07 SE (*n *= 4), respectively.

**Table 3:** Leaf dark respiration rates (*R*_d_, at 30 °C) and ^13^C composition of *R*_d_ after 6 min (*R*_d(6min)_ and $\delta^{ 1 3} {\text{C}}_{{{\text{Rd}}( 6 {\text{min}})}}$; *n *= 4), 3 h (*R*_d(3h)_ and $\delta^{ 1 3} {\text{C}}_{{{\text{Rd}}( 3 {\text{h}})}}$; *n *= 4), and 24 h $\left( {R_{{{\text{d}}( 2 4 {\text{h}})}} \,{\text{and}}\,\delta^{ 1 3} {\text{C}}_{{{\text{Rd}}( 2 4 {\text{h}})}} ;\,n\, = \, 3} \right)$; *n *= 3) determined on *PEPC*-OE versus WT (grown under atmospheric $\delta^{ 1 3} {\text{C}}$ of − 41.6‰) after leaf light exposure at *p*O_2_ of 1.84 kPa

Plant-type	*R*_d(6min)_	*R*_d(3h)_	*R*_d(24h)_	$\delta^{ 1 3} {\text{C}}_{{{\text{Rd}}( 6 {\text{min}})}}$	$\delta^{ 1 3} {\text{C}}_{{{\text{Rd}}( 3 {\text{h}})}}$	$\delta^{ 1 3} {\text{C}}_{{{\text{Rd}}( 2 4 {\text{h}})}}$	$\delta^{ 1 3} {\text{C}}_{\text{dm}}$
	(µmol CO_2_ m^−2^ s^−1^)	(µmol CO_2_ m^−2^ s^−1^)	(µmol CO_2_ m^−2^ s^−1^)	(‰)	(‰)	(‰)	(‰)
*PEPC*–OE	1.61 ± 0.10	1.04 ± 0.07	0.74 ± 0.08	− 42.7 ± 1.54	− 61.6 ± 1.1	− 66.1 ± 0.0	− 61.8 ± 0.3
WT	1.23 ± 0.04	0.77 ± 0.04	0.69 ± 0.02	− 45.2 ± 1.47	− 62.4 ± 1.7	− 67.2 ± 1.0	− 62.3 ± 0.5
Significance	*P* = 0.014	*P* = 0.014	*P* = 0.506	*P* = 0.304	*P* = 0.703	*P* = 0.456	*P* = 0.473

In addition, leaf dry matter ^13^C composition ($\delta^{ 1 3} {\text{C}}_{\text{dm}}$; *n *= 4 for *PEPC*-OE; *n *= 5 for WT) were determined on transgenic and WT plants. Values are mean ± SE. Significance (*P* < 0.05) of the effect of plant-type was evaluated by SAS PROC MIXED

#### Leaf dark respiration responses

**Fig. 4:**
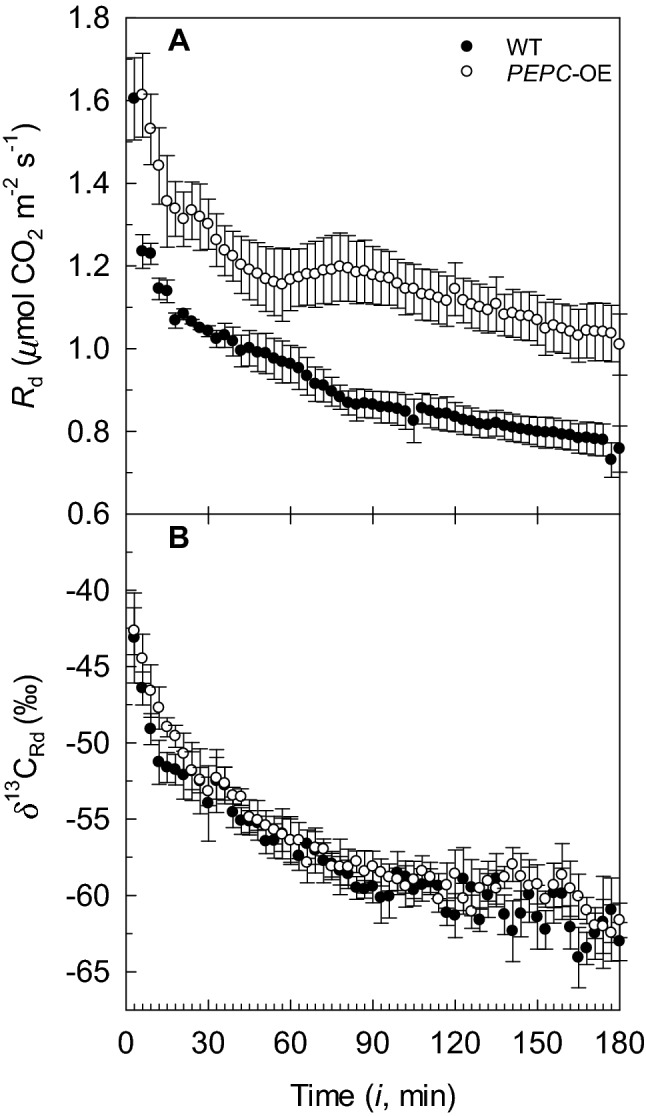
**a** Dynamics of leaf dark respiration rate (*R*_d_) and **b**^13^C composition associated with *R*_d_$\left( {\delta^{ 1 3} {\text{C}}_{\text{Rd}} } \right)$ estimated on *PEPC*-OE (open circles) and WT (closed circles) after leaf exposure to light at *p*O_2_ of 1.84 kPa. Symbols in **a** and **b** correspond to mean value calculated every three min ± SE (*n *= 4)

Before the light–dark transition, the *A* values at *p*O_2_ of 1.84 kPa and PPFD of 750 µmol photons m^−2^ s^−1^ were similar in the *PEPC*-OE and WT plants, with mean rates of 13.9 ± 0.6 SE and 14.1 ± 0.1 SE µmol CO_2_ m^−2^ s^−1^, respectively (*n* = 3). After the light–dark transition, *R*_d_ in the *PEPC*-OE and WT plants had a hyperbolic decrease over the 3-hour interval, with a rapid decline in the first hour (Fig. 4a). The *PEPC*-OE plants showed higher *R*_d_ (~25% enhanced rates; see Fig. 4a; Table 3) compared to WT over the three-hour period, with a statistical significance determined based on the analysis of the three-parameter nonlinear model selected to fit the *R*_d_ responses. Specifically, a significantly greater *R*_d_ lower asymptote was estimated in the *PEPC*-OE *versus* WT plants (µmol CO_2_ m^−2^ s^−1^; *P* < 0.0001), while *R*_d_ ranges (µmol CO_2_ m^−2^ s^−1^; *P* = 1.000) and *R*_d_ exponential rates of change (µmol CO_2_ m^−2^ s^−1^ min^−1^; *P* = 0.114) did not differ between the two plant types (Table S4). The mean values of *R*_L_ inferred from *R*_d(3h),_ were 0.76 ± 0.05 SE for *PEPC*-OE and 0.56 ± 0.03 SE µmol CO_2_ m^−2^ s^−1^ for WT plants.

In the transgenic and WT plants, $\delta^{ 1 3} {\text{C}}_{\text{Rd}}$ after the light–dark transition showed a negative hyperbolic pattern over the three-hour interval, with most of the variations occurring in the first 30 min, and with higher $\delta^{ 1 3} {\text{C}}_{\text{Rd}}$ in the *PEPC*-OE plants compared to WT over the entire dark period (Fig. 4b). The $\delta^{ 1 3} {\text{C}}_{\text{Rd}}$ response in the *PEPC*-OE plants was found to be statistically higher than that in WT plants based on the analysis of the three-parameter nonlinear model selected to fit the $\delta^{ 1 3} {\text{C}}_{\text{Rd}}$ values. In particular, the $\delta^{ 1 3} {\text{C}}_{\text{Rd}}$ ranges were not significantly different (‰; *P* = 0.157), but the $\delta^{ 1 3} {\text{C}}_{\text{Rd}}$ floor value was significantly higher (‰; *P* = 0.003) in the *PEPC*-OE *versus* WT plants (Table S5). However, the values of $\delta^{ 1 3} {\text{C}}_{{( 6 {\text{min}})}} \,{\text{and}}\,\delta^{ 1 3} {\text{C}}_{{( 3 {\text{h}})}}$ were not significantly different between plant types (Table 3). After 24 h in the dark, there were no significant differences in *R*_d(24h)_ and $\delta^{ 1 3} {\text{C}}_{{{\text{Rd}}( 2 4 {\text{h}})}}$ between *PEPC*-OE and WT plants.

## Discussion

### Leaf photosynthetic traits in PEPC-OE versus WT plants

In the current study, the in vitro leaf activity of PEPC was ~ 25 times higher in the *PEPC*-OE plants than that in WT, and the mean PEPC activity relative to Rubisco activity was 79% in the transgenic plants compared to 4% in WT. However, the higher in vitro PEPC activity in the *PEPC*-OE plants had no detectable effect on *A*, or other photosynthetic parameters (e.g., stomatal conductance). Previous studies have also shown no enhancement of *A* in transgenic rice and tobacco expressing *Zm*PEPC, compared to untransformed plants, even though there was a large increase in in vitro PEPC activity (Taniguchi et al. 2008; Hudspeth et al. 1992). While other studies showed that transgenic rice plants expressing *ZmPEPC* had a lower O_2_ sensitivity of *A* compared to WT, due to a decrease in *A* at low O_2_ level (Ku et al. 1999, 2000; Agarie et al. 2002; Fukayama et al. 2003), other studies showed that under photorespiratory conditions *A* was higher in transgenic rice expressing *Zm*PEPC (Jiao et al. 2002) or C_4_ PEPC from sugarcane (Lian et al. 2014). Moreover, transgenic *Arabidopsis thaliana* expressing *Zm*PEPC with a tenfold increase of in vitro PEPC activity showed ~18% higher *A* compared to control plants (Kandoi et al. 2016); similar enhancement of *A* was also seen in *Zm*PEPC transgenic wheat (Hu et al. 2012). These different responses to higher C_4_ PEPC activities in transgenic plants of rice and other C_3_ species highlight the need for further research to clarify the physiological impacts as well as the control of C_4_-PEPC (and in particular *Zm*PEPC) activity in rice leaves.

There are several factors that could limit in vivo C_4_-PEPC activity expressed in a C_3_ plant. For example, 3-phosphoglyceric acid (3-PGA) produced by Rubisco could be used for synthesis of PEP to drive additional PEPC reactions to produce oxaloacetate (OAA) and then malate through malate dehydrogenase (MDH). However, the export of PEP from the chloroplast in the cytosol may limit *Zm*PEPC in rice (Taniguchi et al. 2008; Weber and von Caemmerer 2010). Moreover, *Zm*PEPC in rice is known to be in a dephosphorylated status during the light time, and therefore it operates at reduced rates because of the low affinity for PEP and the feedback (allosteric) inhibition by various metabolites such as malate, aspartate, and glutamate (Vidal and Chollet 1997; Jeanneau et al. 2002). This posttranslational PEPC control is involved in mediating carbon–nitrogen interactions; specifically, forms of PEPC with diminished feedback inhibition may increase carbon flux into organic acids (OAA and malate) and amino acids at the expense of starch and soluble sugars (Rademacher et al. 2002; O’Leary et al. 2011). In two previous studies, a 1.5- to 3-fold increase in leaf malate occurred in the light of transgenic tobacco, potato and rice overexpressing PEPC (Hudspeth et al. 1992; Häusler et al. 1999; Rademacher et al. 2002; Ku et al. 2000; Agarie et al. 2002). However, in the current study on rice, the malate content was not significantly higher in *PEPC*-OE plants compared to WT. The accumulation of leaf malate during the photoperiod in *PEPC*-OE rice plants will depend on relative rates of malate synthesis via PEPC, rates of catabolism of malate by mitochondria, and rates of export of malate outside the leaves. In general, the net rate of leaf CO_2_ assimilation incorporates the CO_2_ fluxes through carboxylation, photorespiration, and light respiration, which are difficult to disentangle with traditional measurements of leaf CO_2_ exchange. However, as discussed below, the combined analysis of leaf–atmosphere CO_2_ exchange and discrimination against ^13^CO_2_ can be a useful tool to gain insights into these various fluxes of CO_2_ within the leaf.

### Leaf net discrimination against ^13^CO_2_ in the light, and net biochemical ^13^CO_2_ discrimination in the PEPC-OE plants

In C_3_ plants, leaf net discrimination against ^13^CO_2_ in the light (∆_o_, ‰) integrates the discrimination against ^13^CO_2_ during CO_2_ diffusion (in both gas and liquid phases) and due to the carboxylation and decarboxylation reactions. In particular, the Rubisco ^13^CO_2_ fractionation (*b*_3_) is 29‰, whereas the PEPC net ^13^CO_2_ fractionation associated with bicarbonate fixation (*b*_4_) is − 5.7‰ (Ubierna and Farquhar 2014; von Caemmerer et al. 2014). Therefore, an increase of the carboxylation by PEPC (*β*) would lower the in vivo ^13^CO_2_ carboxylation fractionation (*b*) and potentially decrease ∆_o_ (Farquhar and Richards 1984; Lanigan et al. 2008; Bickford et al. 2010). In the present study, the values of *b* in the WT were set equal to 29.0‰ at all CO_2_ levels, i.e., there was no carboxylation by rice native PEPC given the negligible in vitro PEPC activity determined in WT plants. The leaf net biochemical discrimination against ^13^CO_2_ (∆_bio_) and *b* were significantly lower by ~2‰ in the *PEPC*-OE compared to WT plants, across the *p*CO_2_ experimental range. Although in *PEPC*-OE plants there was a tendency for ∆_bio_ to decrease with lower *p*CO_2_ (i.e., *β* to increase, in accordance with Abadie and Tcherkez 2018), the CO_2_ dependency was likely minimized due to the low photorespiratory measurement conditions (Leegood and von Caemmerer 1988, 1989, 1994). However, across the *p*CO_2_ experimental interval the calculated mean *β* was ~6% in *PEPC*-OE plants compared to zero in WT (Fig. 2b). The significant change in ∆_bio_ and increase in *β* indicate a contribution of *Zm*PEPC to carboxylation in the *PEPC*-OE plants compared to WT even though there was no detectable difference in *A* between the plant types. This might suggest either that carbon carboxylated by PEPC did not go through Calvin cycle (Häusler et al. 1999) or that Rubisco had a lower carboxylation efficiency in the *PEPC*-OE plants compared to WT (Agarie et al. 2002; Fukayama et al. 2003). The latter explanation seems unlikely in the present study, where slightly higher Rubisco activity was determined in the *PEPC*-OE plants compared to WT, and comparable maximum carboxylation efficiency values (µmol CO_2_ m^−2^ s^−1^ Pa^−1^) were calculated for the two plant types (data not shown). Alternatively, based on the collective information from previous studies, overexpression of PEPC may have enhanced the anaplerotic pathway (Fukayama et al. 2003; Miyao and Fukayama 2003; O’Leary et al. 2011; Kandoi et al. 2016; Abadie and Tcherkez 2018) rather than promoting carbon fixation of the C_4_-like photosynthetic pathway.

The difference in *b* between the *PEPC*-OE and WT plants could in part be affected by the magnitude of $\frac{{R_{\text{L}} e^{*} }}{{\left( {A + R_{\text{L}} } \right)}}$ (that contributes to ∆_bio_mod_ in Eq. 1) in the transgenic plants. However, in the current study, the *R*_L_/(*A* + *R*_L_) ratio was low at all three *C*_a_. In addition, given the relatively low sensitivity of *b* to *R*_L_ (see Fig. S4A), even a higher *R*_L_ in the *PEPC*-OE in comparison to WT plants will have a minor contribution to the ^13^C discrimination analysis.

In the present study, leaf photosynthetic measurements were conducted under low *p*O_2_ (1.84 kPa) to reduce the uncertain contributions of photorespiration and potential re**-**fixation of (photo)respired CO_2_ by PEPC and Rubisco (Ku et al. 2000; Agarie et al. 2001) to leaf net discrimination against ^13^CO_2_, and therefore to minimize the errors in ∆_bio_ estimate. In addition, since low *p*O_2_ reduces the inhibition of *R*_L_ relative to rates of leaf respiration in the dark (*R*_d_) (Abadie et al. 2017; Tcherkez et al. 2017a, b; Gauthier et al. 2018), *R*_L_ was modelled at 25 °C from *R*_d_ at 30 °C after three hours from the light–dark transition. Based on previous studies, there is indication of negative effects exerted by the atmospheric CO_2_ level, in the short-term, on leaf respiration activity in the light (Tcherkez et al. 2008, 2017a). Since the predicted *R*_L_ were applied over all measurement CO_2_ conditions, a potential overestimation of *R*_L_ may have therefore risen at the highest *C*_a_; nevertheless, its effect on the ^13^C discrimination analysis is considered minor given the low *R*_L_/(*A* + *R*_L_) ratio. Since there is no evidence in the literature of different downregulation of *R*_L_ in transgenic *PEPC*-OE compared WT rice the higher *R*_L_ predicted for the *PEPC*-OE compared to WT plants may be due to *Zm*PEPC activity.

In the ∆_bio_ analysis, the *g*_m_ values determined on WT based on leaf net discrimination against ^13^CO_2_ were applied to *PEPC*-OE plants. This assumption of equal *g*_m_ in transgenic and WT plants was supported by nonsignificantly different *g*_m_ determined by leaf ^18^O discrimination (Yakir 1998; Gillon and Yakir 2000; Barbour et al. 2016; Ubierna et al. 2017) on the *PEPC*-OE and WT plants under the same experimental conditions of the present study (Table S2). It is theoretically possible that the increased PEPC activity in the transgenic plants would enhance *g*_m_ compared to WT, as previously reported by Alonso-Cantabrana and von Caemmerer (2016) for a C_3_–C_4_ intermediate species. However, an increase in *g*_m_ would lead to a further decrease in the estimate of ∆_bio_ as a 0.5 µmol CO_2_ m^−2^ s^−1^ Pa^−1^ raise in *g*_m_ lowers ∆_bio_ by ~1‰ (Fig. S3).

Furthermore, in the present study the ∆_bio_ analysis assumed no ^13^CO_2_ respiratory fractionation via TCA cycle (*e*, ‰; Ghashghaie et al. 2003; Werner and Gessler 2011); however, even a large *e* would have exerted a minor effect on *b* and *g*_m_, as presented in Method S5 Fig. S4C and S4D, respectively.

### Leaf dark respiration and ^13^C composition of evolved CO_2_ in PEPC-OE versus WT plants

In the present study, following leaf transition from light to dark, *R*_d_, and $\delta^{ 1 3} {\text{C}}_{\text{Rd}}$ showed a hyperbolic decrease over a three-hour period in both plant types, with a significantly higher *R*_d_ and $\delta^{ 1 3} {\text{C}}_{\text{Rd}}$ in the *PEPC*-OE compared to WT plants (Table S4 and Table S5). It has been previously shown that the high *R*_d_ in the first 30 min after light–dark transition, *light enhanced dark respiration* (LEDR), results primarily from leaf respiration of substrates as organic acids (in particular malate), produced in the prior light period (Werner and Gessler 2011; Tcherkez et al. 2012, 2015, 2016b; Gessler et al. 2017). Based on the analysis conducted on several species, Lehman et al. (2016b) reported how the leaf respiration rate and ^13^C composition of evolved CO_2_ during the LEDR time may be only weakly related, and how changes in both responses are highly species-specific. In the present study, for both plant types, a close correlation between *R*_d_ and $\delta^{ 1 3} {\text{C}}_{\text{Rd}}$ over the three-hour dark period was determined (r > 0.90). However, leaf CO_2_ evolution in the dark and its $\delta^{ 1 3} {\text{C}}_{\text{Rd}}$ composition contain information about leaf metabolism and respiratory substrates (Lehman et al. 2016b). For example, Barbour et al. (2007) and Gessler et al. (2009) reported for castor bean (*Ricinus communis*) that leaf LEDR mainly comes from the decarboxylation of ^13^C heavier metabolites, mostly malate, and that the declines in LEDR and $\delta^{ 1 3} {\text{C}}_{\text{Rd}}$ over time are caused by the decrease in malate availability as respiratory substrate. In the dark, leaf malate can be decarboxylated via malic enzyme in mitochondria to make pyruvate, or alternatively, it can be oxidized to OAA via NAD-MDH (Wiskich and Dry 1985; Douce and Neuburger 1987). Pyruvate and OAA can subsequently be used in anaplerotic reactions to replenish TCA cycle intermediates when they are consumed for lipid or amino acid synthesis (Doubnerová and Ryšlavá 2011; Muramatsu et al. 2014; Lehmann et al. 2015, 2016b). In the current study, the *R*_d_ integral over 30 min after light–dark transition was 0.48 mmol CO_2_ m^−2^ higher in *PEPC*-OE with respect to WT plants. Theoretically, if the enhancement of LEDR in the *PEPC*-OE plants during the first 30 min in the dark was due to malate alone, this would have required total 0.12 mmol malate m^−2^, i.e., ~ the double of the leaf malate content determined during the photoperiod in *PEPC*-OE plants. Since no significant difference in leaf malate accumulation was observed between *PEPC*-OE and WT plants, other leaf organic acids (e.g., fumarate, citrate; Agarie et al. 2002; Abadie and Tcherkez 2018) produced in the light duration may have also contributed to a bigger pool of dark respiratory substrates in the *PEPC*-OE compared to WT (Tcherkez et al. 2012; Lehman et al. 2016b). In addition, part of the leaf malate may be in an inactive pool (e.g., in the vacuole) as observed in C_4_ plants (Hatch 1979; Arrivault et al. 2017) and therefore not readily available for LEDR. Another carbon source for *R*_d_ in the *PEPC*-OE plants could be via function of PEPC in the dark, when *Zm*PEPC has been reported to be in a phosphorylated status (Fukayama et al. 2003; Leegood 2013). In the dark period, by utilizing part of the PEP produced during glycolysis, *Zm*PEPC in rice could lead to the increased synthesis of malate that can be metabolized in the TCA cycle as substrate for anaplerosis during mitochondrial respiration, with the possibility to raise the CO_2_ evolution (Suzuki et al. 2006).

Over the 3 h in the dark, the variations in $\delta^{ 1 3} {\text{C}}_{\text{Rd}}$ for both *PEPC*-OE and WT plants suggest a decrease in the contribution to *R*_d_ of respiratory substrates produced in the leaf chamber (*L*_ch_) before the light–dark transition and a complementary increase in the contribution of substrates produced in the growth chamber. The L_ch_ carbon assimilates were estimated to account from ~50% of the substrates for *R*_d_ after 6 min to ~30% after 30 min, and only ~10% after three hours in the dark, with no differences between transgenic and WT plants (see Fig. S5). Tcherkez et al. (2010) estimated that recent carbon assimilates in sunflower (*Helianthus annuus*) provided 40–60% of substrates for leaf respiration (via a pool with a half-life of several hours) both in the light and in the dark; similar contribution was determined by Nogués et al. (2004) on French bean (*Phaseolus vulgaris*) for approximately two-hour dark after leaf illumination. The higher $\delta^{ 1 3} {\text{C}}_{\text{Rd}}$ responses in *PEPC*-OE plants compared to WT during LEDR may partially depend on a relatively greater contribution to leaf dark respiration of organic acids, which are ^13^C enriched, compared to other respiratory substrates as sugars and amino acids (Lehman et al. 2016b). In particular, during the photoperiod, the PEPC activity in the *PEPC*-OE plants could promote the production of ^13^C-enriched OAA, compared to WT, which can be converted to malate by MDH and used to feed leaf respiration after light–dark transition (Barbour et al. 2007; Gessler et al. 2009; Werner et al. 2011; Lehmann et al. 2016b). In addition, the tendency of a higher $\delta^{ 1 3} {\text{C}}_{{{\text{Rd}}( 2 4 {\text{h}})}} \,\left( {{\text{and}}\,\delta^{ 1 3} {\text{C}}_{\text{dm}} } \right)$ in *PEPC*-OE versus WT plants may indicate that the substrates available for the TCA cycle produced in the G_ch_ had a slightly more enriched ^13^C composition in the transgenic plants compared to WT. This may suggest that in the growth chamber at current atmospheric *p*O_2_ and *p*CO_2_ of 184 Pa, a possible lower leaf net discrimination against ^13^CO_2_ could have occurred in the *PEPC*-OE plants compared to WT. For both plant types, a lower $\delta^{ 1 3} {\text{C}}_{{{\text{Rd}}( 2 4 {\text{h}})}}$ than $\delta^{ 1 3} {\text{C}}_{\text{dm}}$ is in agreement with Tcherkez et al. (2003).

## Conclusions

There are uncertainties in the physiological effect of transgenic expression of *Zm*PEPC in the C_3_ plant rice. However, enhancement of PEPC activity is a key step in engineering C_4_ photosynthesis into C_3_ plants. In the present study, the transgenic rice plants expressing *Zm*PEPC had higher in vitro PEPC activity, a significant fraction of carbon fixed by PEPC and a decreased ∆_bio_ compared to WT (determined at *p*CO_2_ from below to above current ambient level). However, *A* was not significantly different between *PEPC*-OE and WT plants, while *R*_d_ and ^13^C composition of leaf dark-evolved CO_2_ were higher in the *PEPC*-OE plants *versus* WT, additionally indicating enhanced in vivo PEPC activity in the *PEPC*-OE plants. These results suggest that although *Zm*PEPC appears to be functional in the *PEPC*-OE rice plants, there are some factors likely related to substrate availability (PEP and/or bicarbonate) or posttranslational controls (e.g., involving regulatory phosphorylation) that reduce the activity of the enzyme in vivo during the photoperiod. Insights into these limitations may be discernible with detailed analysis of metabolite pools (organic acids, carbohydrates, and starch), ad hoc estimates of *g*_m_, and magnitude of refixation of photorespired CO_2_ compared to WT. This will provide the much needed understanding to further the development of a functioning C_4_ photosynthetic cycle in rice.

## Electronic supplementary material

Below is the link to the electronic supplementary material.

Supplementary material 1 (DOCX 2701 kb)
