# Supplementary material for: Transgenic maize phosphoenolpyruvate carboxylase alters leaf–atmosphere CO2 and 13CO2 exchanges in Oryza sativa
Source: Photosynth Res. 2019 Jul 19;142(2):153–67. doi: 10.1007/s11120-019-00655-4 (PMC6848035; doi:10.1007/s11120-019-00655-4)

**Electronic Supplementary Material**

**Title:** Transgenic maize phospho*enol*pyruvate carboxylase alters leaf-atmosphere CO_2_ and ^13^CO_2_ exchange in *Oryza sativa*

**Photosynthesis Research** xx: xx-xx

**Authors:** Rita Giuliani, Shanta Karki, Sarah Covshoff, Hsiang-Chun Lin, Robert A. Coe, Nuria Koteyeva, Marc A. Evans, W. Paul Quick, Susanne von Caemmerer, Robert T. Furbank, Julian M. Hibberd, Gerald E. Edwards, Asaph B. Cousins

Corresponding author: Asaph B. Cousins, e-mail: acousins@wsu.edu

**Fig. S1** Verification of transgenic rice lines expressing maize *phospho*enol*pyruvate carboxylase*

**Fig. S2** Immunoblot and immunolocalization analysis of leaf PEPC protein

**Method S1** Leaf biochemical analysis

**Table S1** Description of the abbreviations, and symbol and unit of the environmental parameters and leaf variables used in the text

**Method S2** Estimate of leaf net biochemical discrimination against ^13^CO_2_ and its dependency on *g*_m_

**Fig. S3** Dependency of Δ_bio_ on *g*_m_ at different atmospheric CO_2_ levels

**Method S3** Estimate of *g*_m_

**Table S2** Values of *g*_m_ based on leaf net discrimination against ^18^O

**Table S3** Variables and values used to estimate of the fractional contributions of respiratory substrates from L_ch_ and G_ch_ carbon assimilates to *δ*^13^C of dark evolved CO_2_

**Method S4** Statistical analysis

**Table S4** Selection of the model to fit *R*_d_ response

**Table S5** Selection of the model to fit *δ*^13^C_Rd_ response

**Method S5** Description of the sensitivity analysis for *in vivo* ^13^CO_2_ carboxylation fractionation (*b*) and *g*_m_ to both *R*_L_ and ^13^CO_2_ fractionation during decarboxylation in the light (*e'* = *e*+ e*); results are in **Fig.** **S4**

**Fig. S5** Fractional contributions of respiratory substrates from L_ch_ and G_ch_ carbon assimilates to *δ*^13^C of dark evolved CO_2_

**Fig. S1** Verification of transgenic *Oryza sativa* lines expressing maize (*Zea mays*) *phospho*enol*pyruvate carboxylase* (*ZmPEPC*). **(A)** Schematic representation of the intact maize *PEPC* gene used for rice transformation (GenBank, Accession no. X15642). The full genomic sequence was used in transformation: 8,124 base pair (bp) containing all exons, introns and native promoter and terminator. RB and LB are right and left border, respectively. H indicates the HindIII cut site used in DNA blot analysis. The probe (394 bp) indicated by a thin line was used for DNA blot hybridization. **(B)** DNA blot analysis at the T_3_ generation shows that *PEPC* insertion-event-28 carried a single T-DNA insertion and was homozygous, as all eight randomly sampled progeny analyzed inherited the T-DNA. bp is base pair; MW is the molecular weight marker; WT (wild type) is untransformed *Oryza sativa* used as a negative control transgene expression

**
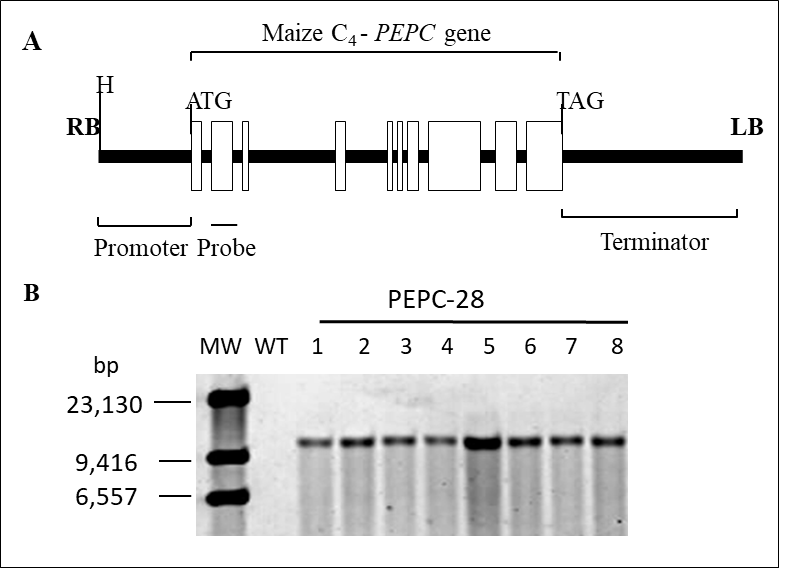
**

**Fig. S2** Immunoblot and immunolocalization analysis of leaf PEPC protein. **(A)** Immunoblot analysis of *Zm*PEPC protein accumulation in *Oryza sativa* leaves from events PEPC-28, PEPC-60, PEPC-62 and PEPC-76 at the mid-tillering plant growth stage of T_3_ generation (*n* = 8). Maize leaves were used as a reference for *Zm*PEPC protein accumulation in the rice leaves. Mean *Zm*PEPC protein accumulation in PEPC-28, PEPC-60, PEPC-62 and PEPC-76 events were 87, 104, 137 and 116% respectively relative to maize, based on equal leaf surface area. No *Zm*PEPC protein accumulation was detected in the untransformed rice (WT) used as a negative control for T-DNA insertion. **(B)** Immunolocalization of *Zm*PEPC protein, shown in yellow, in leaf cells of maize cv. B73, untransformed control rice (WT), transgenic PEPC-28, PEPC-60, PEPC-62 and PEPC-76 events. Chlorophyll fluorescence is shown in red. In the PEPC-28 event, *Zm*PEPC protein detection is localized to the mesophyll (M) cells, while in the other three transgenic rice events there was detectable *Zm*PEPC accumulation in both bundle sheath (BS) and M cells

**A**

Maize

WT

1 2 3 4 5 6 7 8

PEPC-28

100 kDa


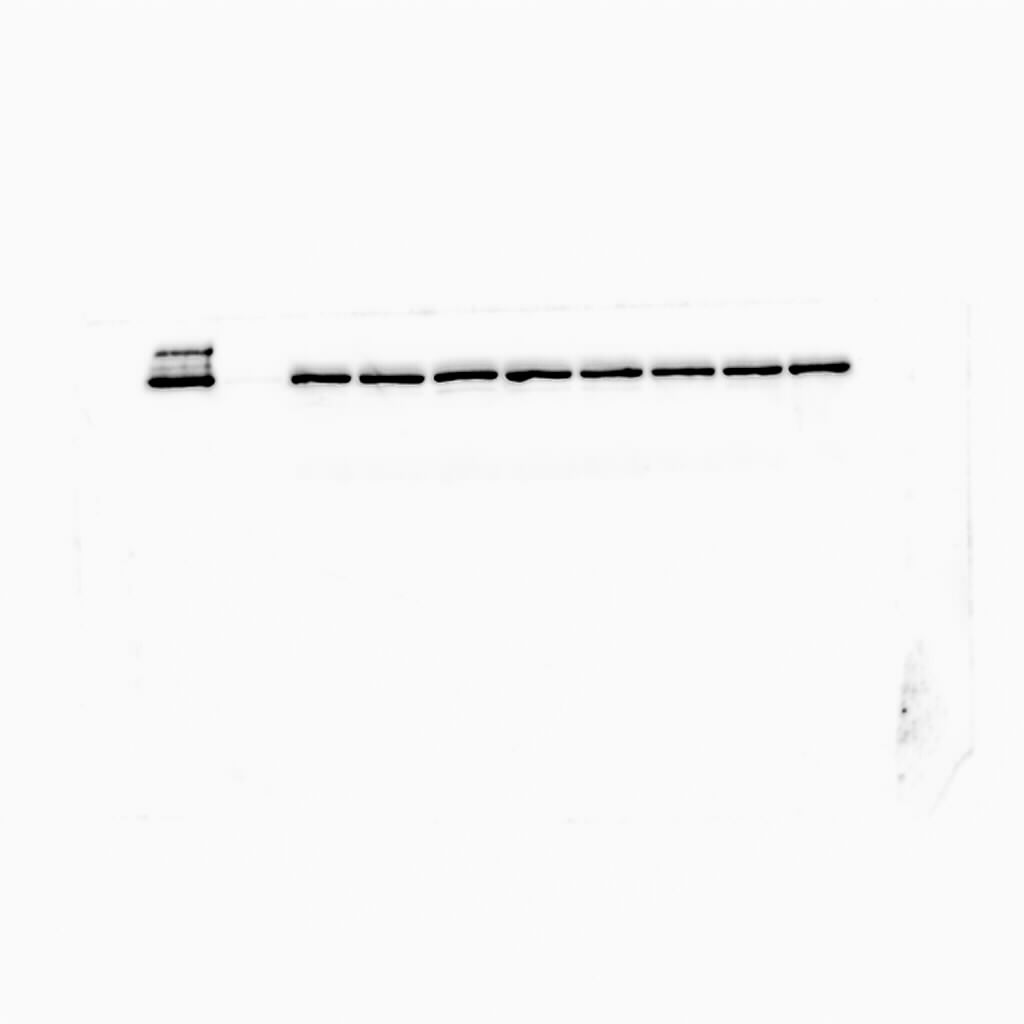


Maize

WT

1 2 3 4 5 6 7 8

PEPC-62


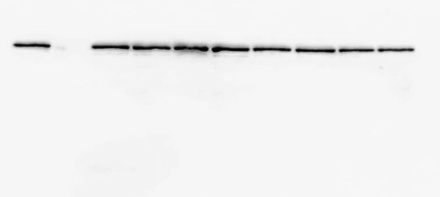


Maize

WT

1 2 3 4 5 6 7 8

PEPC-76


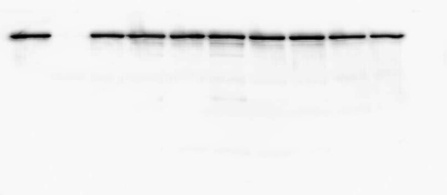


Maize

WT

1 2 3 4 5 6 7 8

PEPC-60


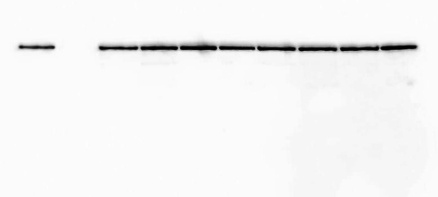

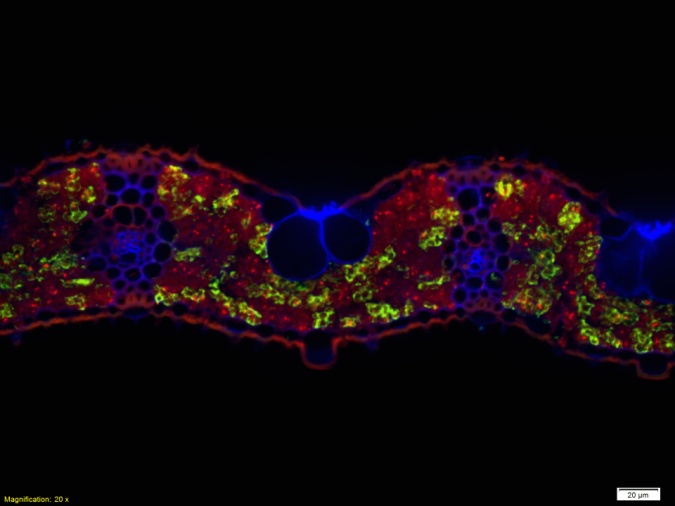


PEPC-28


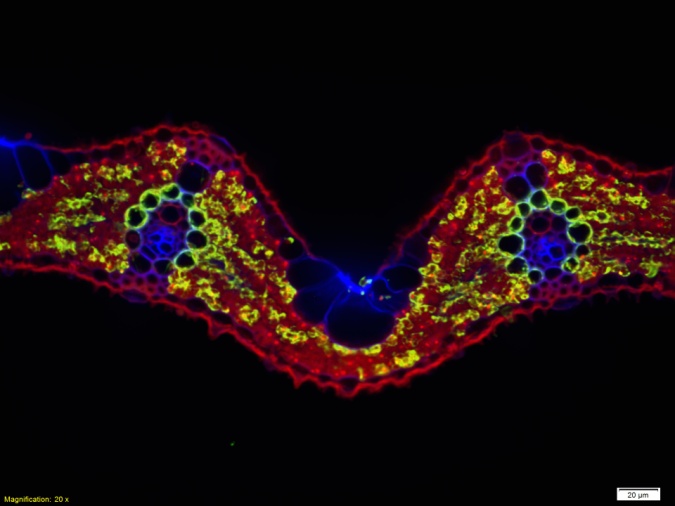


PEPC-62


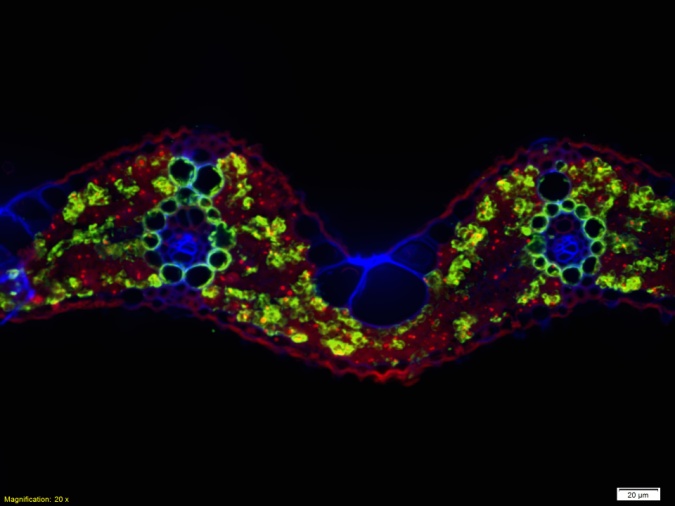


PEPC-60

**B**

BS cell

BS cell

M cell

M cell

BS cell


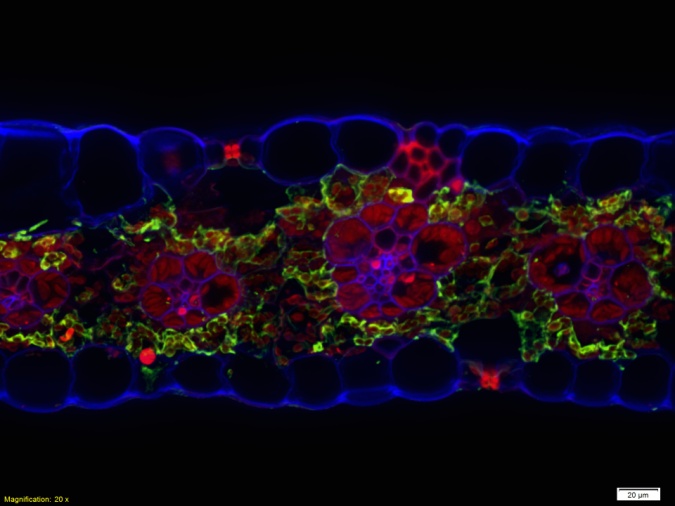


Maize cv. B73


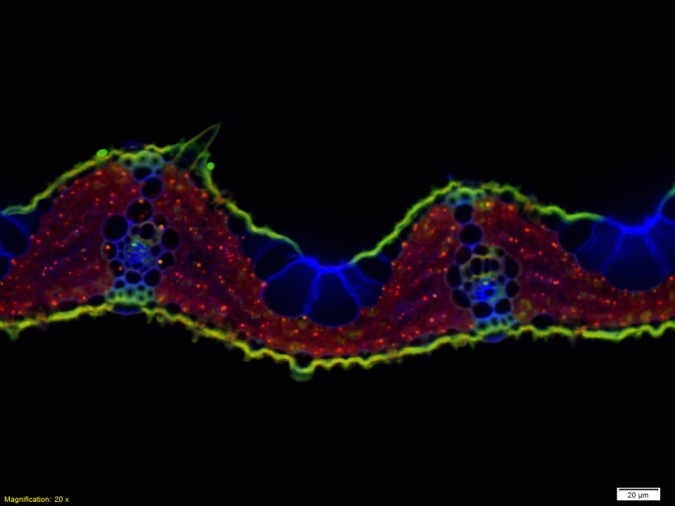


Control Rice


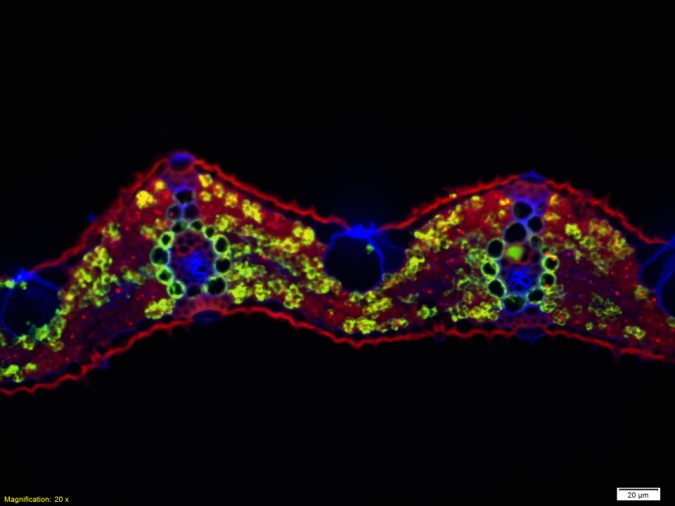


PEPC-76

M cell

BS cell

M cell

M cell

BS cell

M cell

M cell

**Method S1** Leaf biochemical analysis

PEPC *level*

Soluble proteins were extracted from fully expanded leaves of four to five week-old transgenic *PEPC*-OE and WT rice, and maize plants according to Koteyeva *et al.* (2015). For each genotype, two separate protein extractions (biological replicates) were performed, each one using the leaf tissue collected on one plant. Protein concentration was determined with an RC DC protein quantification kit (Bio**-**Rad, Hercules, CA). Protein separation, blotting onto a nitrocellulose membrane and visualization were carried out according to Koteyeva *et al.* (2015). Immunoblots (*n* = 2 for both *PEPC-*OE and WT) were performed using primary antibodies for anti**-***Zea mays* PEPC IgG (1:100 000) (Chemicon, Temecula, CA, USA) raised in rabbit. Band intensities were quantified with ImageJ 1.37 software (NIH, USA). The level of PEPC for both *PEPC-*OE and WT rice was shown as fraction (%) of *Z. mays*.

PEPC *and Rubisco* *activity*

Leaf samples were taken from the mid-section of young leaves on three-week old rice plants, and from the mid to distal portion of fully**-**expanded leaves on four**-**five weeks old rice and maize plants. The older rice leaves were the same used for the photosynthesis measurements. All leaf samples were immediately frozen in liquid N_2_ and stored at −80°C. Leaf sample extracts of *PEPC*-OE and WT rice, and maize plants (*n* = 3) were used to determine the *in vitro* activity of PEPC and Rubisco (expressed per unit leaf surface area; *µ*mol HCO_3_^−^ m^−2^ s^−1^ and *µ*mol CO_2_ m^−2^ s^−1^, respectively) at 25 °C using an Evolution^TM^ 300 UV**-**Vis spectrophotometer (Thermo Scientific, Waltham, MA, USA). Specifically, PEPC activity was determined as previously described by Cousins *et al*. (2007) and Pengelly *et al*. (2010), while Rubisco was determined according to Walker *et al*. 2013.

*Malate content*

The malate content was determined on leaf extracts of *PEPC*-OE and WT rice plants (*n* = 5) by spectrophotometry and expressed per unit leaf surface area (mmol malate m^−2^). The portions of leaves chosen for photosynthesis measurements were sampled immediately after their use and frozen in liquid N_2_. Levels of malate were determined according to Hatch (1979), with modifications by Edwards *et al.* (1982). The reactions, which were coupled to NADP-malic enzyme, were initiated by adding 0.4 mM NADP and the final absorbance change at 340 nm was recorded after two h at 30 °C using a Thermo Scientific spectrophotometer. A known amount of malate was used as a standard for procedure verification.

**References**

Cousins AB, Baroli I, Badger MR, Ivakov A, Lea PJ, Leegood RC, von Caemmerer S ( 2007) The role of phosphoenolpyruvate carboxylase during C_4_ photosynthetic isotope exchange and stomatal conductance. Plant Physiol 145: 1006-1017

Edwards GE, Ku MSB, Hatch MD (1982) Photosynthesis in *Panicum milioides*, a species with reduced photorespiration. Plant Cell Physiol 23: 1185-1195

Hatch MD (1979) Mechanism of C_4_ photosynthesis in *Chloris gayana*: pool sizes and kinetics of ^14^CO_2_ incorporation into 4-carbon and 3-carbon intermediates. Arch Biochem Biophys 194: 117-127

Koteyeva NK, Voznesenskaya EV, Edwards GE (2015) An assessment of the capacity for phosphoenolpyruvate carboxykinase to contribute to C_4_ photosynthesis. Plant Sci 235: 70-80

Pengelly JJL, Sirault XRR, Tazoe Y, Evans JR, Furbank RT, von Caemmerer S (2010) Growth of the C_4_ dicot *Flaveria bidentis*: photosynthetic acclimation to low light through shifts in leaf anatomy and biochemistry. J Exp Bot 61: 4109-4122

Walker B, Ariza LS, Kaines S, Badger MR, Cousins AB (2013) Temperature response of *in vivo* Rubisco kinetics and mesophyll conductance in *Arabidopsis thaliana*: comparisons to *Nicotiana tabacum*. Plant Cell Environ 36: 2108-2119

| **Table S1** Description of the abbreviations, and symbol and unit of the environmental parameters and leaf variables used in the text | | | | | | | |  |
| --- | --- | --- | --- | --- | --- | --- | --- | --- |
| **ABBREVIATION** | **DESCRIPTION** |  | | | | | |  |
| CA | Carbonic anhydrase |  | | |  |  |  |  |
| G_ch_ | Growth chamber |  | | |  |  |  |  |
| h | Hour |  | | |  |  |  |  |
| HCO_3_^−^ | Bicarbonate |  | | |  |  |  |  |
| L_ch_  LEDR | Leaf chamber  Light enhanced dark respiration |  | | |  |  |  |  |
| M | Mesophyll |  | | |  |  |  |  |
| MDH | Malate dehydrogenase |  | | |  |  |  |  |
| min | Minute |  | | |  |  |  |  |
| NAD | Nicotinamide adenine dinucleotide |  | | |  |  |  |  |
| NADP  OAA | Nicotinamide adenine dinucleotide phosphate  Oxaloacetate |  | | |  |  |  |  |
| PEP | Phospho*enol*pyruvate |  | | |  |  |  |  |
| PEPC | PEP carboxylase enzyme |  | | |  |  |  |  |
| *PEPC*-OE | *PEPC* overexpressing |  | | |  |  |  |  |
| Rubisco | Ribulose-1,5**-**bisphosphate carboxylase/oxygenase |  | | |  |  |  |  |
| RuBP | Ribulose-1,5**-**bisphosphate |  | | |  |  |  |  |
| TCA | Tricarboxylic acid |  | | |  |  |  |  |
| *Zm*PEPC | *Zea mays* C_4_-PEPC enzyme |  | | |  |  |  |  |
| *ZmPEPC* | *Zea mays* C_4_-*PEPC* gene |  | | |  |  |  |  |
| **SYMBOL** | **ENVIRONMENTAL PARAMETERS/ LEAF VARIABLES** | | | **UNIT** | | |  |  |
|  |  |  | | | |  |  |  |
| *A* | Net CO_2_ assimilation rate per unit (one side) leaf surface area | | *µ*mol CO_2_ m^-2^ s^-1^ | | | | | |
| *a’* | Cumulative (air) ^13^CO_2_ fractionation during CO_2_ diffusion through boundary layer and stomata | | ‰ | | | | | |
| *a_l_* | ^13^CO_2_ fractionation during CO_2_ diffusion in the liquid phase | | ‰ | | | | | |
| *b* | *In vivo* ^13^CO_2_ carboxylation fractionation | | ‰ | | | | | |
| *b*_3_ | Rubisco ^13^CO_2_ fractionation | | ‰ | | | | | |
| *b*_4_ | Net PEPC ^13^CO_2_ fractionation | | ‰ | | | | | |
| *b_s_* | ^13^CO_2_ fractionation as CO_2_ enters solution | | ‰ | | | | | |
| *C*_a_ | CO_2_ molar fraction or CO_2_ partial pressure set in the leaf chamber | | *µ*mol CO_2_ mol^-1^ air; Pa | | | | | |
| *C*_c_ | CO_2_ molar fraction or CO_2_ partial pressure in the chloroplast | | *µ*mol CO_2_ mol^-1^ air; Pa | | | | | |
| *C*_i_ | CO_2_ molar fraction or CO_2_ partial pressure in the intercellular air space | | *µ*mol CO_2_ mol^-1^ air; Pa | | | | | |
| *e* | ^13^CO_2_ fractionation due to respiration *via* TCA cycle | | ‰ | | | | | |
| *e** | Experimental ^13^CO_2_ fractionation associated with *R*_L_ | | ‰ | | | | | |
| *f* | Photorespiratory ^13^CO_2_ fractionation | | ‰ | | | | | |
| *g*_m_ | Mesophyll conductance to CO_2_ diffusion from the substomatal cavity to chloroplast stroma | | *µ*mol CO_2_ m^-2^ s^-1^ Pa^-1^ | | | | | |
| *g*_sC_ | Stomatal conductance to CO_2_ diffusion | | *µ*mol CO_2_ m^-2^ s^-1^ Pa^-1^ | | | | | |
| *p*CO_2_ | Partial pressure of atmospheric CO_2_ | | Pa | | | | | |
| *p*O_2_ | Partial pressure of atmospheric O_2_ | | kPa | | | | | |
| PPFD | Photosynthetic Photon Flux Density | | *µ*mol photons m^-2^ s^-1^ | | | | | |
| *R*_d_ | Leaf dark respiration rate per unit (one side) leaf surface area | | *µ*mol CO_2_ m^-2^ s^-1^ | | | | | |
| *R*_d(24h)_ | *R*_d_ at 24 h after light-dark transition | | *µ*mol CO_2_ m^-2^ s^-1^ | | | | | |
| *R*_d(3h)_ | *R*_d_ at 3 h after light-dark transition | | *µ*mol CO_2_ m^-2^ s^-1^ | | | | | |
| *R*_d(6min)_ | *R*_d_ at 6 min after light-dark transition | | *µ*mol CO_2_ m^-2^ s^-1^ | | | | | |
| *R*_L_ | Light mitochondrial non-photorespiratoy respiration rate per unit leaf surface area | | *µ*mol CO_2_ m^-2^ s^-1^ | | | | | |
| *t* | Correction factor for ternary effects | | ‰ | | | | | |
| *t*_leaf_ | Leaf temperature | | °C | | | | | |
| VPD | Vapor Pressure Deficit | | kPa | | | | | |
| *β* | Fraction of carboxylation by PEPC | | mol C_(by PEPC)_ mol^-1^ C_(by Rubisco+PEPC)_ | | | | | |
| Δ^13^C_mod_ | Leaf net discrimination against ^13^CO_2_ in the light predicted based on Ubierna and Farquhar (2014) | | ‰ | | | | | |
| Δ_bio_ | Leaf net biochemical discrimination against ^13^CO_2_ | | ‰ | | | | | |
| Δ_bio_mod_ | Δ_bio_predicted according to Griffiths *et al.* (2007) | | ‰ | | | | | |
| Δ_e_ | ^13^CO_2_ discrimination associated with mitochondrial non**-**photorespiratory respiration | | ‰ | | | | | |
| Δ_f_ | ^13^CO_2_ discrimination associated with photorespiration | | ‰ | | | | | |
| Δ_gm_ | ^13^CO_2_ discrimination associated with mesophyll conductance to CO_2_ diffusion | | ‰ | | | | | |
| Δ_i_ | ^13^CO_2_ discrimination associated with carboxylation, boundary layer and stomatal CO_2_ diffusion | | ‰ | | | | | |
| Δ__ | Observed (instantaneous) leaf net discrimination against ^13^CO_2_ in the light | | ‰ | | | | | |
| ** | CO_2_ compensation point | | Pa | | | | | |
| * *^*^ | CO_2_ compensation point in absence of mitochondrial non**-**photorespiratory respiration | | Pa | | | | | |
| *δ*_in_ | *δ* ^13^C of CO_2_ entering the leaf chamber | | ‰ | | | | | |
| *δ*_out_ | *δ* ^13^C of CO_2_ leaving the leaf chamber | | ‰ | | | | | |
| ^Rd^G_ch_substr_ | Fractional contribution of respiratory substrates from G_ch_ carbon assimilates to *δ*^13^C of dark evolved CO_2_ | | ‰/‰ | | | | | |
| ^Rd^L_ch_substr_ | Fractional contribution of respiratory substrates from L_Ch_ carbon assimilates to *δ*^13^C of dark evolvedCO_2_ | | ‰/‰ | | | | | |
| * *^13^C | ^13^Ccomposition of CO_2_ | | ‰ | | | | | |
| * *^13^C_dm_ | ^13^C signature of leaf dry matter | | ‰ | | | | | |
| * *^13^C_Gch_ | * *^13^C of CO_2_ in the growth chamber during the light period | | ‰ | | | | | |
| * *^13^C_Lch_Ph_ | Representative*  *^13^C of carbon assimilates produced in the L_ch_ | | ‰ | | | | | |
| *δ* ^13^C_Rd_ | *δ* ^13^C of CO_2_ evolved by leaves in the dark | | ‰ | | | | | |
| *δ* ^13^C_Rd(24h)_ | *δ* ^13^C of CO_2_ evolved by leaves after 24 h dark | | ‰ | | | | | |
| *δ* ^13^C_Rd(3h)_ | *δ* ^13^C of CO_2_ evolved by leaves after three h dark | | ‰ | | | | | |
| *δ* ^13^C_Rd(6min)_ | *δ* ^13^C of CO_2_ evolved by leaves after six min dark | | ‰ | | | | | |

**Method S2** Estimate of leaf net biochemical ^13^CO_2_ discrimination and its dependency on *g*_m_

The leaf net biochemical ^13^CO_2_ discrimination (Δ_bio_, ‰) was determined in *PEPC*-OE plants at *C*_a_ of 18.4, 35.0, and 92.1 Pa according to Alonso-Cantabrana and von Caemmerer (2016), based on the model by Griffiths *et al.* (2007) and including a correction for ternary effects (Farquhar and Cernusak 2012) as

$\Delta_{\mathrm{bio}}=\frac{\Delta_{o}-\frac{1}{1-t}a^{'}\left( 1-\frac{C_{i}}{C_{a}} \right)-\frac{1+t}{1-t}\left( a_{l}+b_{s} \right)\frac{A}{g_{m}C_{a}}}{\frac{1+t}{1-t} \frac{1}{C_{a}}\left( C_{i}-\frac{A}{g_{m}} \right)}$ (S1)

where Δ_o_ is the leaf net discrimination against ^13^CO_2_ in the light; *t* is a correction factor for ternary effects; *a'*is the cumulative ^13^CO_2_ fractionation during CO_2_ diffusion through boundary layer and stomata calculated as in Alonso-Cantabrana and von Caemmerer (2016); *C*_i_ and *C*_a_ are intercellular and atmosphere molar fractions (*μ*mol CO_2_ mol^−1^ air); *a*_l_ is the ^13^CO_2_ fractionation during CO_2_ diffusion in the liquid phase (0.7‰); *b*_s_ is the ^13^CO_2_ fractionation as CO_2_ enters solution (1.1‰); *A* is the leaf net CO_2_ assimilation rate; *g*_m_ (mol CO_2_ m^−2^ s^−1^) is the mesophyll CO_2_ conductance determined on WT plants using the procedure in Method S3. The dependency of Δ_bio_ on *g*_m_ in eq. S1, at different atmospheric CO_2_ levels, is displayed in Fig. S3.

**Fig. S3** Dependency of Δ_bio_ on *g*_m_ in eq. S1 at different atmospheric CO_2_ levels (*C*_a_). Mean *A*= 14.4, 27.0 and 35.8 *µ*mol CO_2_ m^−2^s^−1^ were used for *C*_a_= 18.4, 35.0, 92.1 Pa, respectively (the *A* values were as in Table 1 for *PEPC*-OE plants). The arrows indicate the mean Δ_bio_ calculated for *PEPC*-OE plants at the three *C*_a_


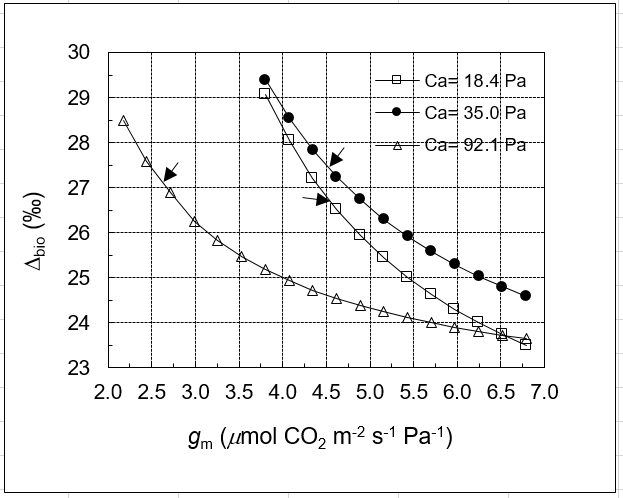


**References**

Alonso-Cantabrana H, von Caemmerer S (2016) Carbon isotope discrimination as a diagnostic tool for C_4_ photosynthesis in C_3_-C_4_ intermediate species. J Exp Bot 67: 3109-3121

Farquhar GD, Cernusak LA (2012) Ternary effects on the gas exchange of isotopologues of carbon dioxide. Plant Cell Environ 35: 1221-1231

Griffiths H, Cousins AB, Badger MR, von Caemmerer S (2007) Discrimination in the dark. Resolving the interplay between metabolic and physical constraints to phosphoenolpyruvate carboxylase activity during the crassulacean acid metabolism cycle. Plant Physiol 143: 1055-1067

**Method S3** Estimate of *g*_m_

Leaf mesophyll conductance to CO_2_ diffusion from intercellular air space to the chloroplast stroma (*g*_m_, *μ*mol CO_2_ m^−2^ s^−1^ Pa^−1^) was determined at *p*O_2_ of 1.84 kPa on WT plants based on Evans and von Caemmerer (2013). The ^13^CO_2_ carboxylationfractionation factor *b* was set equal to the Rubisco ^13^CO_2_ fractionation factor (*b*_3_, 29.0‰) given a negligible *in vitro* PEPC activity determined in WT (see Results). It was assumed that at the same *t*_leaf_ there was no difference between leaf mitochondrial respiration rate in the light (*R*_L_) and three h after light-dark transition (*R*_d(3h)_, *µ*mol CO_2_ m^−2^ s^−1^). *R*_L_ at *t*_leaf_ of 25 °C was therefore predicted from *R*_d(3h)_at 30 °C using the temperature response function in Bernacchi *et al.* (2001). According to Tazoe *et al*. (2009), Evans and von Caemmerer (2013), and Alonso-Cantabrana and von Caemmerer (2016), no ^13^CO_2_ discrimination due to respiration *via* TCA cycle (*e*, ‰) was applied. In addition, under the assumption that all substrates feeding *R*_L_ were from carbon fixed in the growth chamber, an experimental (non-enzymatic; Evans and von Caemmerer 2013; Gong *et al*. 2015; Alonso-Cantabrana and von Caemmerer 2016) ^13^C fractionation associated with *R*_L_ (*e**, ‰) was calculated as e*=*δ*_in_−*δ*^13^C_Gch_, where *δ*_in_ and *δ*^13^C_Gch_ are ^13^C composition (‰) of CO_2_ entering the leaf chamber and in the growth chamber, respectively. Most probably, *δ*_out_ would have been closer than *δ*_in_ to the representative * *^13^C of leaf chamber atmosphere; however, the variation in *e** using *δ*_out_ had been evaluated having a negligible effect (data not shown) on the present leaf photosynthetic ^13^CO_2_ discrimination analysis. Among the input parameters, the CO_2_ photorespiratory compensation point (*Γ* ^*^, *µ*mol CO_2_ mol^−1^ air) was modeled at *t*_leaf_ of 25 °C according to Bernacchi *et al.* (2001), while the photorespiratory fractionation *f* was set equal to 16.2‰ based on Evans and von Caemmerer (2013). The chloroplast *p*CO_2_ (*C*_c_, Pa) was calculated for both *PEPC*-OE and WT plants at the different *C*_a_ levels by Fick’s first law (Nobel 2009) as *C*_c_=*C*_i_−*A*/*g*_m_.

**References**

Alonso-Cantabrana H, von Caemmerer S (2016) Carbon isotope discrimination as a diagnostic tool for C_4_ photosynthesis in C_3_-C_4_ intermediate species. J Exp Bot 67: 3109-3121

Bernacchi CJ, Singsaas EL, Pimentel C, Portis Jr AR, Long SP (2001) Improved temperature response functions for models of Rubisco-limited photosynthesis. Plant Cell Environ 24: 253-259.

Evans JR, von Caemmerer S (2013) Temperature response of carbon isotope discrimination and mesophyll conductance in tobacco. Plant Cell Environ 36: 745-756

Gong XY, Schäufele R, Feneis W, Schnyder H (2015) ^13^CO_2_/^12^CO_2_ exchange fluxes in a clamp‐on leaf cuvette: disentangling artefacts and flux components. Plant Cell Environ 38: 2417-2432

Nobel PS. 2009. Physicochemical and Environmental Plant Physiology. 4^th^ Ed. Academic Press

Tazoe Y, von Caemmerer S, Badger MR, Evans JR (2009) Light and CO_2_ do not affect the mesophyll conductance to CO_2_ diffusion in wheat leaves. J Exp Bot 60: 2291-2301

**Table S2** Values of *g*_m_ based on leaf net discrimination against ^18^O (*g*_m_18O_)determined for *PEPC*-OE and WT plants at different *C*_a_. Values are mean ± SE (*n* = 3).A non-significant plant type effect was determined on *g*_m_18O_ by a PROC MIXED procedure, with Plant-type (*PEPC*-OE and WT) and CO_2_ level as fixed factors and leaves as random factor nested within Plant-type (α = 0.05). In addition, orthogonal contrasts tested the linear and quadratic polynomials for a quantitative relationship between *g*_m_18O_ values and unequal *C*_a_ increments

| Plant type | *C*_a_ | *g*_m_18O_ |
| --- | --- | --- |
|  | (Pa) | (*μ*mol CO_2_ m^−2^ s^−1^ Pa^−1^) |
| *PEPC-*OE | 18.4 | 6.5 ± 0.8 |
|  | 35.0 | 5.4 ± 0.5 |
|  | 92.1 | 1.3 ± 0.2 |
| WT | 18.4 | 5.7 ± 0.8 |
|  | 35.0 | 4.6 ± 0.4 |
|  | 92.1 | 1.9 ± 0.5 |
| Significance | Plant type | **P= 0.478** |
|  | *C*_a_ | P 0.0001 |
|  | POC linear | P= 0.337 |
|  | POC quadratic | P  0.0001 |
|  | Plant type * *C*_a_ | P= 0.908 |
|  |  |  |

**Table S3** Variables and values used to estimate the fractional contributions of leaf chamber (L_ch_) and growth chamber (G_ch_) carbon assimilates to *δ*^13^Cof leaf dark evolved CO_2_ in *PEPC*-OE and WT plants following leaf photosynthesis under *p*O_2_ of 1.84 kPa and *C*_a_ of 35.0 Pa. The variables for computations are *δ*^13^C of CO_2_ in the L_ch_ (*δ*_out_), leaf net discrimination against ^13^CO_2_ in the light (Δ_o_),^13^C discrimination associated to *R*_L_ (Δ_e_) before light-dark transition, ^13^C signature of carbon assimilates produced in the L_ch_ (*δ*^13^C_Lch_Ph_; modeled as *δ*^13^C_Lch_Ph_ = *δ*_out_ – Δ_o_ + Δ_e_), ^13^C composition of dark evolved CO_2_ after 24 h dark (*δ*^13^C_Lch(24h)_), and difference between *δ*^13^C_Lch_Ph_ and *δ*^13^C_Lch(24h)_. The data originate from leaf measurements with *δ*^13^C in CO_2_ entering the L_ch_ (*δ*_in_) enriched compared to G_ch_ (*δ*^13^C_Gch_), and *e** = *δ*_in_ − *δ*^13^C_Gch_ was calculated. For both plant types, the mean for each variable is shown (*n* = 4).

| Plant-type | *δ*^13^C_Gch_ | *δ*_in_ | *e* | *δ*_out_ | Δ_o_ | Δ_e_ | *δ*^13^C_Lch_Ph_ | *δ*^13^C_Lch(24h)_ | *δ*^13^C_Lch_Ph_−*δ*^13^C_Lch(24h)_ |
| --- | --- | --- | --- | --- | --- | --- | --- | --- | --- |
|  | (‰) | (‰) | (‰) | (‰) | (‰) | (‰) | (‰) | (‰) | (‰) |
| *PEPC*-OE | −41.6 | −6.0 | 35.6 | −3.6 | 14.1 | 3.0 | −20.7 | −66.1 | 45.4 |
| WT | −41.6 | −5.0 | 36.6 | −2.8 | 14.2 | 2.3 | −19.3 | −67.2 | 47.9 |

**Method S4** Statistical analysis

Statistical analyses were performed using SAS version 9.4 (SAS Institute, Cary, NC, USA). A PROC MIXED procedure, with Plant-type (*PEPC*-OE and WT) and CO_2_ level as fixed factors and leaves as random factor nested within Plant-type was run to describe the variability of the following leaf traits: *A*, *g*_sC_, *C*_i_, *C*_i_/*C*_a_, *C*_c_, *C*_c_/*C*_a_. Δ_o_ and Δ_bio_ (α = 0.05). In addition, orthogonal contrasts tested the linear and quadratic polynomials for a quantitative relationship between leaf trait values and unequal *C*_a_ increments. A PROC MIXED procedure was applied as One-way ANOVA with Plant-type as the only fixed factor to describe the variability of the following traits: malate content, total N content* * *R*_d(6m)_, *δ*^13^C_Rd(6m)_, *R*_d(3h)_, *δ*^13^C_Rd(3h)_, *R*_d(24h)_* *^13^C_Rd(24h)_ and *δ*^13^C_dm_. One-way ANOVA with CO_2_ level as fixed factor was applied to describe the variability of *g*_m_ in the WT. An ARTool (Aligned Rank Transform for nonparametric factorial ANOVA; Wobbrock *et al*. 2011) analysis in R software environment (R Core team 2013) was applied with Plant-type (*PEPC*-OE and WT) and CO_2_ level as fixed factors and leaves as random factor nested within Plant-type to test the variation of *b* and *β* calculated for the *PEPC*-OE plants compared to constant *b* and *β* values in WT (α = 0.05). For each plant type, with reference to the data in Fig. 3, a linear regression analysis (Zar 2009; α = 0.05) was used to fit the predicted Δ^13^C_mod_ values to the observed Δ_o_ (model II), and then to fit the Δ_o_ values (model I) including an extra value of 4.4‰ at *C*_c_/*C*_a_ = 0; the slopes of the regression lines fitting the Δ_o_ values for the two plant types were compared.

SAS Proc NLMixed was used to fit a von Bertalanffy nonlinear model (Paine *et al.* 2012) to the *R*_d_ and *δ*^13^C_Rd_ response data that were determined for the *PEPC*-OE and WT plant types over a period of 195 min (*n* = 4). The model represents an exponentially decreasing function with parameters for each of the two plant types. The model contains a total of six-parameters and is defined as

$y=(i_{\mathrm{WT}})\left[ \theta_{11}e^{-\theta_{12}x}+\theta_{13} \right]+(i_{PEPC-OE})\left[ \theta_{21}e^{-\theta_{22}x}+\theta_{23} \right]+\varepsilon$ (S2)

The response *y* represents either *R*_d_ or *δ*^13^C_Rd_, *x* is time in three min steps, the first term in brackets represents that portion of the model for WT and the second term in brackets is for *PEPC*-OE, with indicator variables i_WT_ and i*_PEPC-_*_OE_ coding for the WT and *PEPC*-OE treatments, respectively; *ε* is the error. The parameters, *θ*_ij_, have indices i = {1,2} coding for the plant types, with 1 = WT and 2 = *PEPC*-OE and j = {1, 2 and 3} coding for the model parameters, with 1 = range or difference between the y value at time = 0 and the lower asymptote, 2 = exponential rate of change and 3 = the lower asymptote or floor of the mean response.

To assess whether the mean response for the WT and *PEPC*-OE treatments differed, likelihood ratio chi-square tests (G^2^) were developed and a 0.05 level of significance was used for all tests. Four hypotheses were assessed: 1) whether the nonlinear curves differed between the WT and *PEPC*-OE treatments, with null hypothesis *θ*_11_ = *θ*_21_, *θ*_12_ = *θ*_22_ and *θ*_13 =_ *θ*_23_, 2) whether the ranges differed between WT and *PEPC*-OE treatments, with null hypothesis *θ*_11_ = *θ*_21_, 3) whether the exponential rate of change differed between WT and *PEPC*-OE treatments, with null hypothesis *θ*_12_ = *θ*_22_ and 4) whether the asymptotes differed between WT and *PEPC*-OE treatments, with null hypothesis *θ*_13 =_ *θ*_23_. Based on these tests, the most appropriate model structure was to be selected. The results of the model selection for *R*_d_ and *δ*^13^C_Rd_ are shown in Tables S4 and S5, respectively.

**Table S4** Results of the likelihood ratio testing and model selection for *R*_d_. Likelihood-ratio chi-square test (G^2^) was computed for each hypothesis and compared to χ^2^ distribution value with degrees of freedom given as df and level of significance α = 0.05. The results for test 1 indicate that at least one of the parameters differs between the models for WT and *PEPC*-OE (P value < 0.0001). Tests 2 and 3 indicate that no significant difference can be found between the range for WT and *PEPC*-OE (P value = 1.000), nor between the exponential rate of change for WT and *PEPC*-OE (P value = 0.114). However, the hypothesis for test 4 is rejected (P value < 0.0001) and indicates that the lower asymptotes significantly differ between the models for WT and *PEPC*-OE. Based on the testing procedure, the simplest model that fits the data well (best model) is the model that sets *θ*_11_ = *θ*_21_, *θ*_12_ = *θ*_22_, but retains *θ*_13_ and *θ*_23_ as separate parameters. This is also the model selected by Bayesian Information Criterion (BIC) selected model

| Test | Null Hypothesis Tested | G^2^ | df | P value | Best model |
| --- | --- | --- | --- | --- | --- |
| 1 | *θ*_11_ = *θ*_21_, *θ*_12_ = *θ*_22_,*θ*_13 =_ *θ*_23_ | 472.8 | 3 | < 0.0001 |  |
| 2 | *θ*_11_ = *θ*_21_ \| *θ*_12_, *θ*_13_,*θ*_22,_ *θ*_23_ | 0.0 | 1 | 1.000 | *θ*_11_ = *θ*_21_ = 0.482 |
| 3 | *θ*_12_ = *θ*_22_ \| *θ*_11_, *θ*_13_,*θ*_21,_ *θ*_23_ | 2.5 | 1 | 0.114 | *θ*_12_ = *θ*_22_ = 0.019 |
| 4 | *θ*_13_ = *θ*_23_ \| *θ*_11_, *θ*_12_,*θ*_21_,*θ*_22_ | 23.0 | 1 | **< 0.0001** | *θ*_13_ = **0.780**; *θ*_23_ = **1.045** |

**Table S5** Results of the likelihood ratio testing and model selection for *δ*^13^C_Rd_. Likelihood-ratio chi-square test (G^2^) was computed for each hypothesis and compared to χ^2^ distribution value with degrees of freedom given as df and level of significance α = 0.05. The results indicate that at least one of the parameters differs between the models for WT and *PEPC*-OE (P value < 0.0001). Test 2 indicates that no significant difference can be found between the range for WT and *PEPC*-OE (P value = 0.157), while test 3 indicates that there is a significant difference between the exponential rate of change for WT and *PEPC*-OE (P value = 0.029). The null hypothesis for test 4 is rejected (P value = 0.003) and indicates that the lower asymptotes significantly differ between the models for WT and *PEPC*-OE. Based on the testing procedure, the simplest model that fits the data well (best model) is the model that sets *θ*_11_ = *θ*_21_, but retains *θ*_12_, *θ*_22_, *θ*_13_ and *θ*_23_ as separate parameters. This is also the model selected by Bayesian Information Criterion (BIC) selected model

| Test | Null Hypothesis Tested | G^2^ | df | P value | Best model |
| --- | --- | --- | --- | --- | --- |
| 1 | *θ*_11_ = *θ*_21_, *θ*_12_ = *θ*_22_,*θ*_13 =_ *θ*_23_ | 19.0 | 3 | < 0.0001 |  |
| 2 | *θ*_11_ = *θ*_21_ \| *θ*_12_, *θ*_13_,*θ*_22,_ *θ*_23_ | 2.0 | 1 | 0.157 | *θ*_11_ = *θ*_21_ = 16.97 |
| 3 | *θ*_12_ = *θ*_22_ \| *θ*_11_, *θ*_13_,*θ*_21,_ *θ*_23_ | 4.8 | 1 | **0.029** | *θ*_12_ = 0.016 *θ*_22_ = 0.023 |
| 4 | *θ*_13_ = *θ*_23_ \| *θ*_11_, *θ*_12_,*θ*_21,_ *θ*_22_ | 8.7 | 1 | **0.003** | *θ*_13_ = **−62.9**; *θ*_23_ = **−60.5** |

**References**

Paine CET, Marthews TR, Vogt DR, Purves D, Rees M, Hector A, Turnbull LA (2012) How to fit nonlinear plant growth models and calculate growth rates: an update for ecologists. Methods Ecol Evol 3: 245–256

R Core Team (2013) R: a language and environment for statistical computing. R Foundation for Statistical Computing, Vienna, Austria. URL http://www.R-project.org/

Wobbrock JO, Findlater L, Gergle D, Higgins JJ (2011) The aligned rank transform for nonparametric factorial analyses using only ANOVA procedures. Proceedings of the SIGCHI Conference on Human Factors in Computing Systems, Vancouver, BC, CA. ACM Press, New York, pp 143-146

**Method S5** Description of the sensitivity analysis for *in vivo* ^13^CO_2_ carboxylation fractionation (*b*) and *g*_m_ to both *R*_L_ and ^13^CO_2_ fractionation during decarboxylation in the light (*e'* = *e*+ e*); results are in **Fig.** **S4**

Given *b* as the *in vivo* ^13^CO_2_ carboxylation fractionation calculated in *PEPC*-OE plants by combined analysis of eq. S1 and eq. 1, a negative linear dependency of *b* on *R*_L_ from 0 to 2.5 *µ*mol CO_2_ m^-2^ s^-1^ was determined. In particular, a relatively low sensitivity of *b* to *R*_L_ was displayed at *C*_a_ of 35.0 and 92.1 Pa, while a ~ two-fold higher sensitivity was displayed at *C*_a_ of 18.4 Pa (Fig. S4A). A negligible sensitivity of *g*_m_ (determined based on Evans and von Caemmerer 2013) to *R*_L_ was found at all *C*_a_ (Fig. S4B).

In the present study, it was assumed that substrates for *R*_L_ were composed by carbon assimilates produced in the G_ch_ and no ^13^CO_2_ fractionation occurred during respiration *via* TCA pathway. Vice-versa, under the assumption of a ^13^CO_2_ fractionation during *R*_L_ *via* TCA pathway (*e*, ‰), the ^13^CO_2_ fractionation associated with *R*_L_ (*e'*, ‰) would have been *e'* = *e** + *e* as in Kromdijk *et al.* (2010) and von Caemmerer *et al*. (2014), based on Wingate *et al*. (2007), where *e** is the difference between *δ*^13^C of the CO_2_ entering the L_ch_ and in the G_ch_. A positive linear dependency of *b* on *e* from 0 to −10‰was determined; in particular, a low sensitivity of *b* to *e* was displayed at *C*_a_ of 35.0 and 92.1 Pa, while ~ two-times higher sensitivity was displayed at *C*_a_ of 18.4 Pa (Fig. S4C). A negligible sensitivity of *g*_m_ to *e* was found at all *C*_a_ levels (Fig. S4D). The trends of the sensitivity of *b* and *g*_m_ to *e'* are also reported in Fig. S4E and S4F, respectively, which mimicked, as expected, the sensitivity of *b* and *g*_m_ to *e*.

**References**

Kromdijk J, Griffiths H, Schepers HE (2010) Can the progressive increase of C_4_ bundle sheath leakiness at low PFD be explained by incomplete suppression of photorespiration? Plant Cell Environ33: 1935-1948

von Caemmerer S, Ghannoum O, Pengelly JJL, Cousins AB (2014) Carbon isotope discrimination as a tool to explore C_4_ photosynthesis. J Exp Bot 65: 3459-3470

Wingate L, Seibt U, Montcrieff JB, Jarvis PG, Lloyd JOL (2007) Variations in ^13^C discrimination during CO_2_ exchange by *Picea sitchensis* branches in the field. Plant Cell Environ 30: 600-616

**Fig. S4** Sensitivity of *b* and *g*_m_ to *R*_L_, *e* and *e'*. In all plots, the squares (□) refer to *C*_a_ of 18.4 Pa, circles (●) to *C*_a_ of 35.0 Pa, and triangles (∆) to *C*_a_ of 92.1 Pa. Sensitivity of **(A**) *b* and **(B)** *g*_m_ to *R*_L_ from 0 to 2.5 *µ*mol CO_2_ m^−2^ s^−1^; the dashed vertical line in **(A)** identifies the *b* value in correspondence of the *R*_L_ predicted for the *PEPC*-OE plants. Sensitivity of **(C)** *b* and **(D)** *g*_m_ to *e* from 0 to −10‰. Sensitivity of **(E)** *b* and **(F)** *g*_m_ to *e'* from −6.4 to −16.4‰, as generated by *e* from 0 (as in the present study) to −10‰ and *e** equal to −6.4‰ (difference between *δ*_in_ = −48.0‰ and *δ*^3^C_Gch_ = −41.6‰). In plots **(A)**, **(C)**, and **(E)** the *b* values at the three *C*_a_ were determined based on the corresponding mean *A*, *C*_i_, *C*_i_/*C*_a_ and Δ_o_ values on *PEPC*-OE plants. In plots **(B)**, **(D)** and **(F)** the *g*_m_ values at the three *C*_a_ were determined based on the corresponding *A*, *C*_i_, *C*_i_/*C*_a_ and Δ_o_ on WT plants. In plots **(C)** and **(E)** the mean values of *R*_L_ for *PEPC*-OE plants were used for calculations, while in plots **(D)** and **(F)** the mean values of *R*_L_ for WT plants were applied


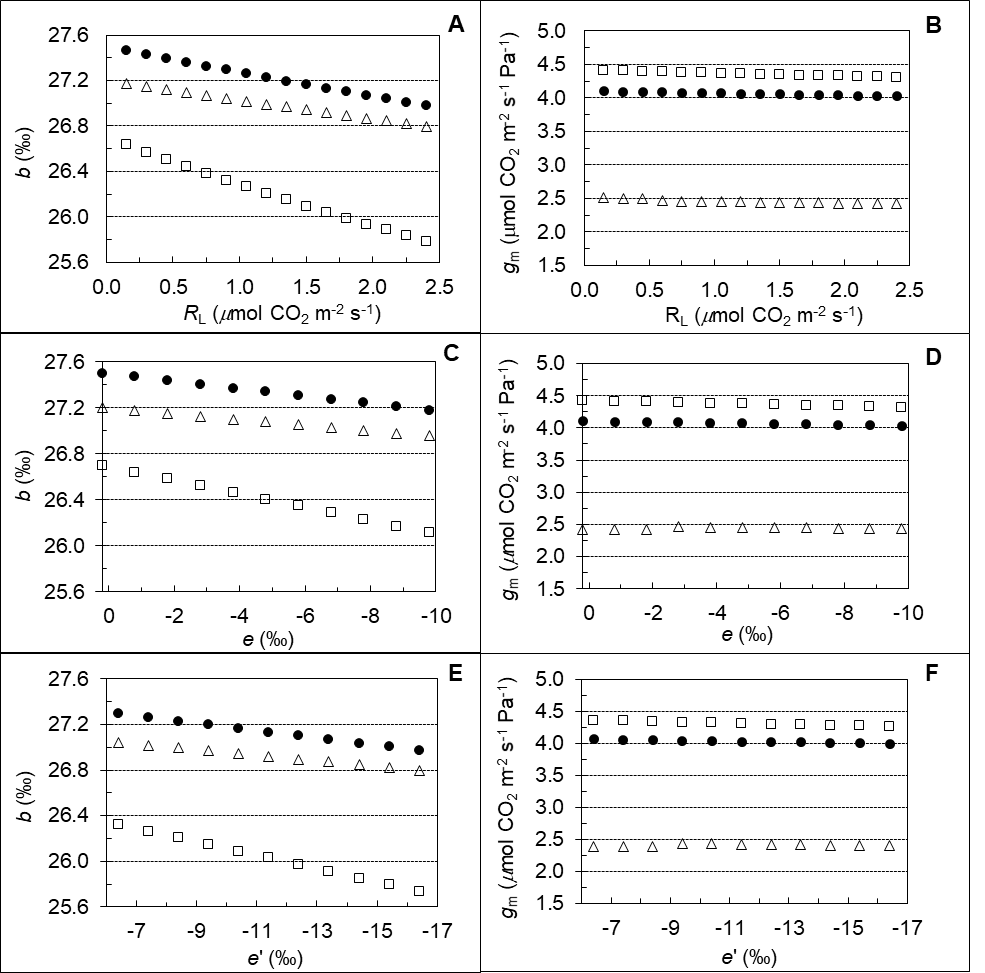


**Fig. S5** Fractional contributions of respiratory substrates from carbon assimilates produced in the L_ch_ and G_ch_ to *δ*^13^C of dark evolved CO_2_. In the dark, over 195 min by *i* steps of three min from light-dark transition, fractional contributions of recent L_ch_ assimilates (^Rd^L_chsubstr(_*_i_*_)_, ‰/‰) and G_ch_ assimilates (^Rd^G_chsubstr(_*_i_*_)_, ‰/‰) to *δ*^13^C_Rd(_*_i_*_)_ for *PEPC-*OE (open circles) and WT (closed circles) plants. Total fractional contributions of L_ch_ and G_ch_ assimilates to *δ*^13^C_Rd(_*_i_*_)_ is equal to 1.0.Symbols correspond to mean values calculated every three min (*n* = 4). Dashed and continuous (mostly overlapping) lines represent logarithmic trend lines (R^2^ > 0.90) for *PEPC*-OE and WT, respectively.


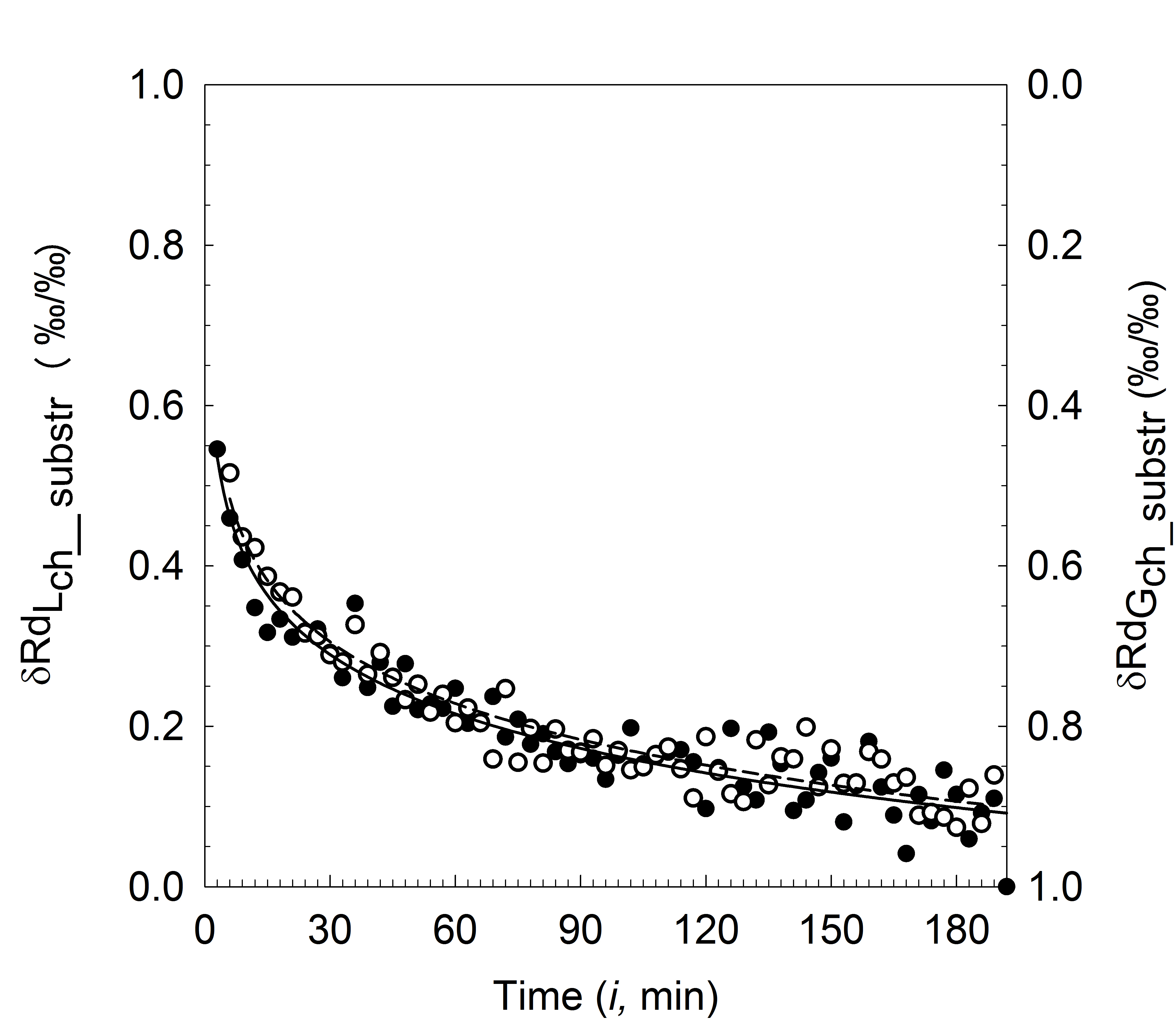

Supplement: Supplementary file 1 — Supplementary material 1 (DOCX 2701 kb) [file 11120_2019_655_MOESM1_ESM.docx]
